# Topological isomers of a potent wound healing peptide: Structural insights and implications for bioactivity

**DOI:** 10.1016/j.jbc.2025.110340

**Published:** 2025-06-04

**Authors:** Tiziano Raffaelli, David T. Wilson, Mehdi Mobli, Michael J. Smout, Guangzu Zhao, Rozita Takjoo, Paramjit S. Bansal, Rilei Yu, Zixuan Zhang, Alex Loukas, Norelle L. Daly

**Affiliations:** 1 Australian Institute of Tropical Health and Medicine, James Cook University, Cairns, Australia; 2 Centre for Advanced Imaging, The University of Queensland, Queensland, Australia; 3 Key Laboratory of Marine Drugs, Chinese Ministry of Education, School of Medicine and Pharmacy, Ocean University of China, Qingdao, China; 4 Laboratory for Marine Drugs and Bioproducts, Qingdao Marine Science and Technology Center, Qingdao, China

**Keywords:** mini-granulin fold, isomer, disulfide-rich peptides, oxidative folding, cell proliferation

## Abstract

There are numerous examples of topological isomers in organic chemistry, but such isomers are rare in disulfide-rich peptides. Here, we characterize two structurally well-defined topological isomers in a peptide (GRN-P4A) containing the mini-granulin fold. The mini-granulin fold is emerging as an important disulfide-rich structural motif with promising implications for the enhancement of wound-healing strategies. The two topological isomers of GRN-P4A have well-defined structures that do not interconvert, and although they have the same disulfide bond connectivity and similar overall structures, they have structural differences related to the first inter-cysteine loop. These structural changes influence the bioactivity as the isomers have significant differences in their cell proliferation activity. Prediction of the structure using AlphaFold3 identified the correct disulfide bond connectivity, but the structure of Loop 1 was similar to the less abundant isomer of GRN-P4A and did not indicate topological isomerization. These topological isomers introduce significant complexity to the understanding of folding mechanisms in this class of peptides, and potentially other disulfide-rich peptides, offering valuable insights for protein design and engineering by presenting a novel topological fold-switching mechanism. Additionally, they hold practical implications to produce GRN-P4A, given its promising potential as a wound-healing agent.

Disulfide-rich peptides, characterized by multiple disulfide bonds, have emerged as promising candidates for therapeutic applications due to their exceptional structural stability and diverse biological activities (1). These peptides, often derived from natural sources such as plants and the venoms of spiders, scorpions, and cone snails, demonstrate significant potential in biomedical, structural, and pharmaceutical research (2, 3, 4, 5, 6, 7). Of particular importance from a drug discovery perspective is ziconotide (SNX-111; Prialt), a calcium channel blocker, approved by the U.S. Food and Drug Administration in 2005 for the treatment of chronic pain (8).

While solid-phase peptide synthesis (SPPS) is widely employed for peptide preparation (9), the synthesis of disulfide-rich peptides remains challenging (10), primarily due to the complexity of the oxidative folding process that governs the proper formation of disulfide bonds. The correct fold of disulfide-rich peptides is directly linked to their biological activity, with only one well-structured configuration typically corresponding to the native, active form (11). However, oxidative folding involves a complex mechanism in which disulfide bonds form and rearrange, often resulting in multiple intermediate states and unstructured isomers (12, 13). This complexity arises from the numerous possible disulfide connectivity patterns that can form during the oxidative folding process, increasing factorially with the number of cysteine residues present in the peptide (14).

The folding landscape of disulfide-rich peptides is further complicated by the presence of topological isomers, which exhibit identical disulfide connectivity but differ in the spatial arrangement of their residues. These differences lead to distinct three-dimensional structures that can significantly impact biological activity (15). In practice such topological isomers have rarely been characterized and a thorough understanding of the formation of these isomers, as well as the ability to control their production, is crucial for harnessing the full therapeutic potential of disulfide-rich peptides.

In this study, we investigated the folding mechanism and biological activity of GRN-P4A, a 24-residue peptide containing three disulfide bonds previously engineered from the liver-fluke granulin family growth factor, *Ov*-GRN-1 (16). The peptide has potent activity in cell proliferation and animal models of wound healing, with these *in vitro* and *in vivo* assays correlating well. Detailed analysis of the peptide oxidation process resulted in the formation of four isomers, with variable bioactivity. Of particular interest are the two isomers that eluted first during reversed-phase high-performance liquid chromatography (RP-HPLC) analysis, and displayed well-defined structures with identical disulfide connectivity, representing topological variants of GRN-P4A. The two isomers have a mini-granulin fold (17) and are characterized by a highly knotted disulfide system involving two stacked lariats. Here we demonstrate that these isomers adopt two distinct conformations that cannot interconvert, likely due to restricted rotation around proline 2, which is crucial for maintaining their structural integrity. This hypothesis is further supported by the synthesis of a GRN-P4A mutant in which proline 2 was replaced with glycine, resulting in the absence of isomerism following oxidation. These findings offer valuable insights into the oxidative folding mechanisms of disulfide-rich peptides and provide a foundation for the design of more structurally stable and potent *Ov*-GRN-1-derived peptides for therapeutic use as wound healing agents.

## Results

### GRN-P4A oxidation and purification

**Figure 1:**
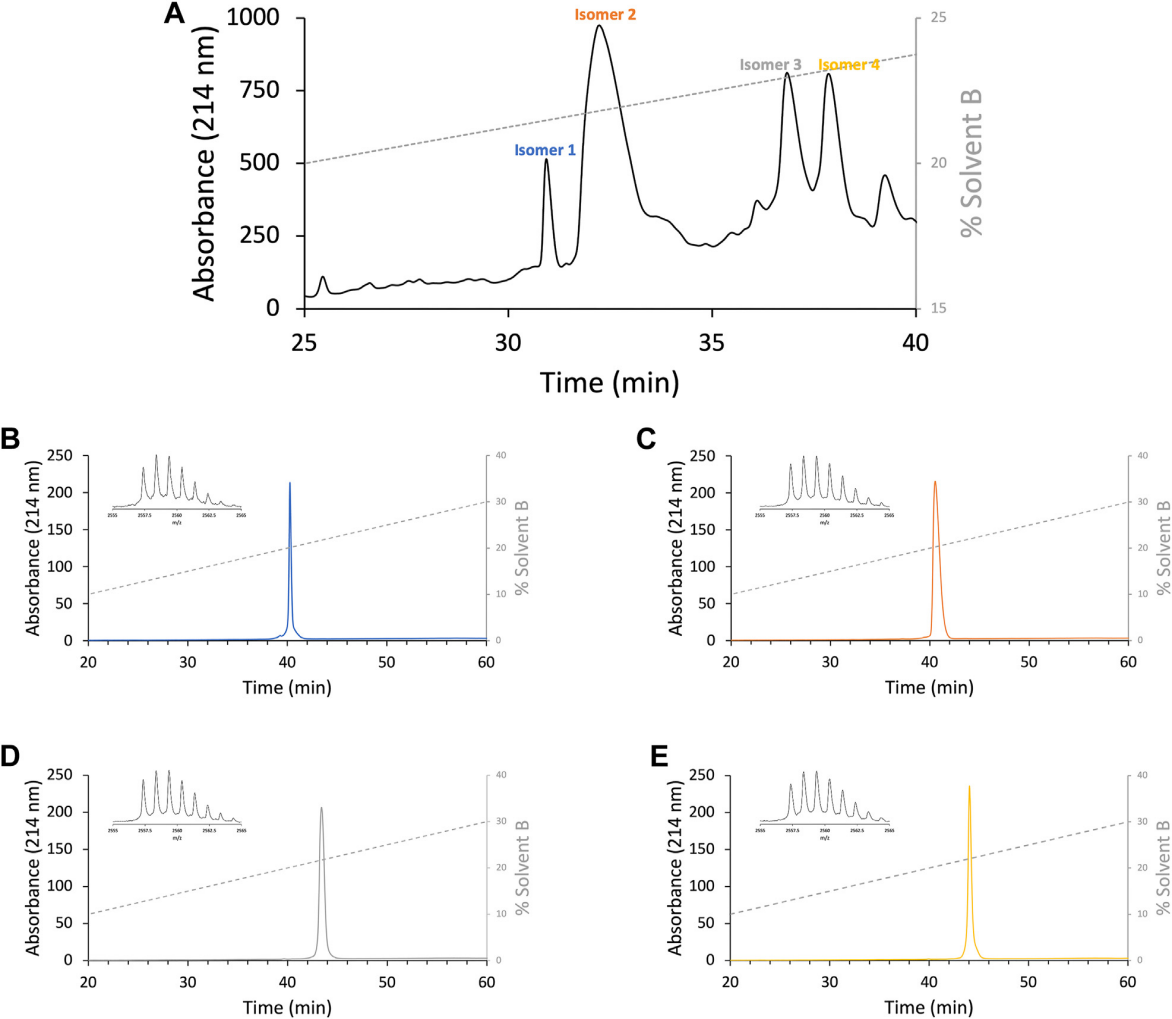
**RP-HPLC chromatogram of oxidized GRN-P4A with analytical traces of purified isomers and MALDI-MS spectra**. *A*, Analytical RP-HPLC profile of fully oxidized GRN-P4A, displaying four peaks corresponding to isomer 1 to 4, *left* to *right* respectively. *B*, analytical RP-HPLC of purified GRN-P4A isomer 1 with elution time of 40.2 min. Inset: MS spectrum of the compound [M + H]^+^ at *m/z* 2557.4 (theoretical [M + H]^+^ = 2558.0 *m/z*, error 234.6 ppm). *C*, analytical RP-HPLC of purified GRN-P4A isomer 2 with elution time of 40.6 min. Inset: MS spectrum of the compound [M + H]^+^ at *m/z* 2557.5 (theoretical [M + H]^+^ = 2558.0 *m/z*, error 195.5 ppm). *D*, analytical RP-HPLC of purified GRN-P4A isomer 3 with elution time of 43.4 min. Inset: MS spectrum of the compound [M + H]^+^ at *m/z* 2557.4 (theoretical [M + H]^+^ = 2558.0 *m/z*, error 234.6 ppm). *E*, analytical RP-HPLC of purified GRN-P4A isomer 4 with elution time of 44.1 min. Inset: MS spectrum of the compound [M + H]^+^ at *m/z* 2557.4 (theoretical [M + H]^+^ = 2558.0 *m/z*, error 234.6 ppm).

Linear GRN-P4A was chemically synthesized *via* Fmoc SSPS and oxidized to facilitate disulfide bond formation as described in the Experimental Procedures. The oxidation reaction resulted in four predominant isomers, which were isolated using RP-HPLC, and their masses were analyzed through MALDI-TOF mass spectrometry. Based on the mass analysis, all of these peaks corresponded to fully oxidized forms of GRN-P4A. These four oxidation products are referred to as isomer 1 through isomer 4, based on their elution order, with isomer 1 eluting first and isomer 4 eluting last. Notably, isomer 2 constitutes the predominant product (≈60%) of the oxidation reaction, whereas isomers 3 and 4 were poorly resolved from each other and eluted approximately 7 min after isomer 1, the sharpest and lowest intensity peak (Fig. 1, *A–E*). Specifically, isomer 1 eluted at 30.8 min with 21.5% solvent B, followed by isomer 2 at 32.2 min with 21.8% solvent B. Isomers 3 and 4 eluted at 37.0 and 38.0 min, respectively, with solvent B concentrations of 22.9% and 23.2%. Tandem mass spectrometry (MS/MS) analysis generated comparable fragmentation patterns for isomers 1 and 2, as well as for isomers 3 and 4 (Fig. S1), suggesting potential structural similarities between these pairs of peptides, as fragmentation patterns can reflect underlying structural features (18). The impact of structural features on fragmentation patterns is highlighted in the MS/MS fragmentation pattern of the linear form of GRN-P4A which contained most of the b- and y-ions in contrast to the disulfide bond isomers (Fig. S1).

### Structural analysis by NMR spectroscopy

The structural conformations of the purified peptides were analyzed using NMR spectroscopy, offering valuable insights into the folding behavior of the different isomers. The one-dimensional spectra of isomers 1 and 2 showed good dispersion in the amide region, whereas isomers 3 and 4 had significant overlaps in this region, indicating a lack of distinct chemical environments for the amide protons. This observation is consistent with the TOCSY (total correlation spectroscopy) spectra, where isomers 1 and 2 displayed clear and sharp cross-peaks, indicative of well-folded peptides with β-sheet elements. In contrast, the TOCSY spectra of isomers 3 and 4 showed less well-resolved cross-peaks and overlapping spin systems suggesting more dynamic or disordered structures (Fig. S2). The similarities in isomers 1 and 2, and isomers 3 and 4, are also reflected in the behavior of the peptides in size-exclusion chromatography (Fig. S3).

**Figure 2:**
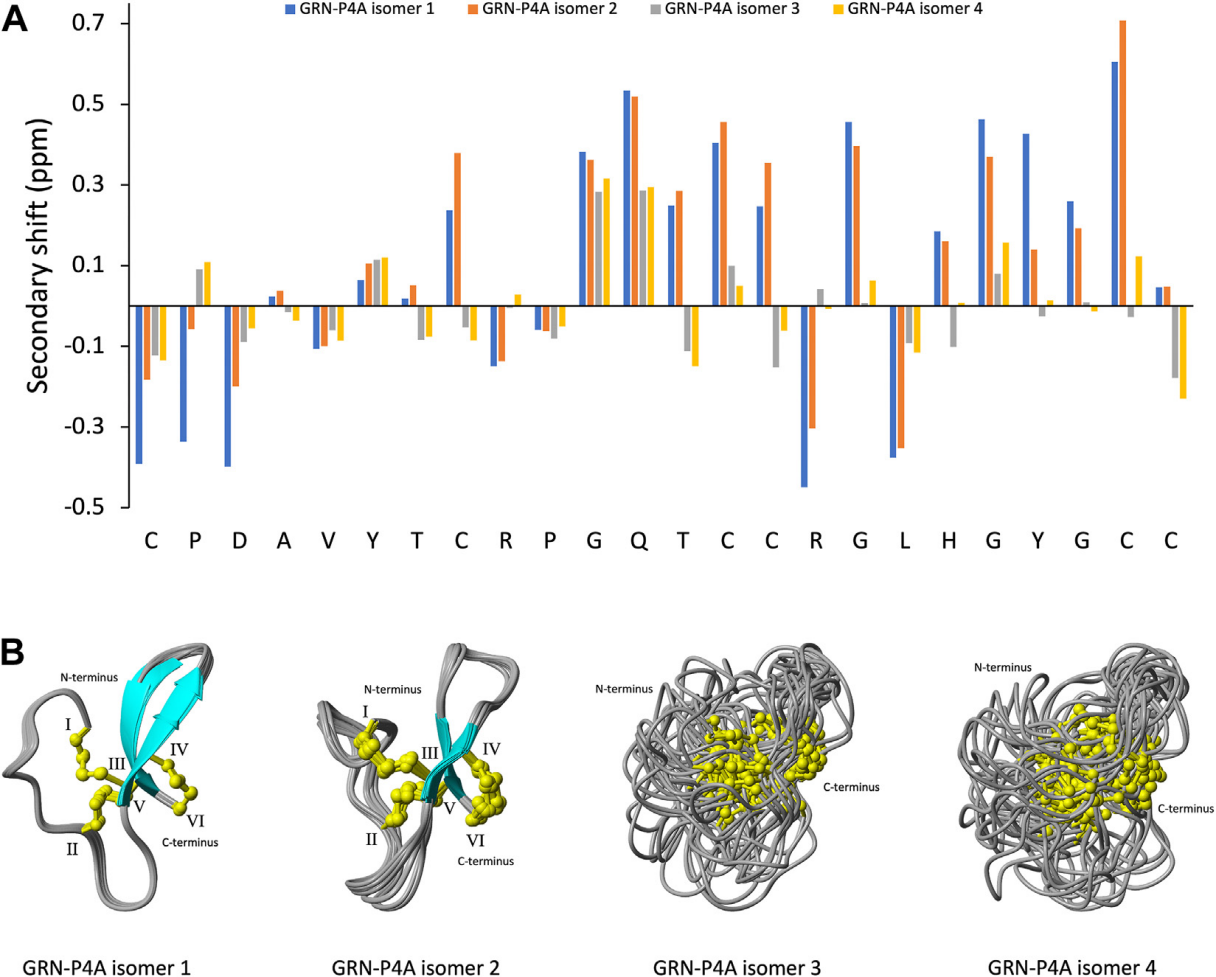
**Comparison of secondary shifts and ensemble of the 20 three-dimensional structures with the lowest target function for GRN-P4A isomers**. *A*, the secondary shifts were obtained by subtracting random coil shifts (19) from the observed αH shifts. GRN-P4A peptides display comparable secondary shift trends between isomers 1 and 2, and between isomers 3 and 4. Notably, isomers one and two exhibit very similar secondary shifts, with the exception of residues 1, 2, 3, and 21, where a prominent distinction is observed at Pro^2^. A continuous stretch of five consecutive positive shifts between Gly^11^ and Cys^15^, as well as six positive shifts between His^19^ and Cys^24^, suggests the presence of conserved, well-defined structural elements, potentially β-sheets. In contrast, isomers 3 and 4 show only seven positive αH shifts which are distributed throughout the sequence. This distribution suggests a more dynamic or disordered structure. *B*, the structures of GRN-P4A were elucidated using NMR spectroscopy, which confirmed that isomers 1 (PDB code: 9MXF) and 2 (PDB code: 9MXE) adopt well-defined conformations, characterized by a conserved granulin disulfide bond connectivity (Cys^I^-Cys^III^, Cys^II^-Cys^V^, and Cys^IV^-Cys^VI^). In contrast, isomers 3 and 4 exhibit more dynamic structures with the same cysteine connectivities but lack β-sheet elements. The cysteine residues are denoted with Roman numerals (I-VI), while disulfide bonds are represented in *yellow* (with the four *yellow*-colored atom representing the β-methylene group (-CH_2_-) and the sulfur atom (-S-) of each cysteine residue involved in the formation of the disulfide bridge). β-sheets are shown in *cyan* for structural distinction.

Secondary shifts, calculated by subtracting random coil values (19) from the observed αH shifts, revealed comparable trends within isomers 1 and 2, and within isomers 3 and 4. The analysis indicated the absence of β-sheet structures in isomers 3 and 4, as only seven positive αH secondary shifts were observed, with considerable distribution throughout the sequence. In contrast, GRN-P4A isomers 1 and 2 displayed nearly 70% positive secondary shifts, corresponding to 16 residues. Among these, a continuous stretch of five consecutive positive shifts between glycine 11 and cysteine 15, along with six positive shifts between histidine 19 and cysteine 24, suggests the presence of a conserved, well-defined β-sheet region that may contribute to the stability and folding of the peptide (Fig. 2*A*). Key chemical shift differences between isomers 1 and 2 were observed at residues 1, 2, 3, and 21, with the most significant difference at proline 2. In isomer 2, the secondary shift for proline 2 was approximately six times greater than in isomer 1, indicating significant conformational variation at this position. This marked difference suggests a localized structural rearrangement that could impact the overall folding of the peptide or alter interactions with nearby residues or regions.

The analysis of two-dimensional spectra, including COSY, NOESY, TOCSY, and nitrogen (^15^N) and carbon (^13^C) heteronuclear single quantum coherence (HSQC), ensured unequivocal determination of resonance shifts for most of the backbone amide groups, carbons and sidechain protons, providing further insights into the structural differences between the isomers. These assignments allowed three-dimensional structures to be calculated using CYANA, incorporating the 15 possible disulfide bond connectivities for each isomer. This method has been previously used to analyze disulfide-rich peptides such as cyclotides and conotoxins (17, 20, 21). NOE (nuclear Overhauser effect) data and dihedral angle constraints were utilized to generate initial structural models. χ1 (CHI1) angle restraints derived from exclusive correlation spectroscopy spectral analysis (22) and hydrogen bond restraints were then incorporated. The hydrogen bonds were predicted based on the preliminary structures and amide protons with slow exchange in deuterium exchange experiments (Fig. S4). Analysis of αδ NOEs indicated that proline 2 and proline 10 adopt a *trans* conformation in each isomer. This conclusion is further supported by the Cβ and Cγ chemical shifts of proline 2 and proline 10, as determined by [^1^H, ^13^C]-HSQC. When the difference between the Cβ and Cγ chemical shifts is between 0 and 4.8 ppm, the peptide bond conformation is predicted to be 100% in the *trans* conformation. By contrast, when it is in the range of 9.15 to 14.4 ppm it is 100% predicted to be in a *cis* conformation (23). In all four isomers the differences between the proline Cβ and Cγ chemical shifts is less than 4.8 ppm, with the exception of proline two in isomer 1 where the difference is 7.03 ppm and therefore is in the ambiguous range (Fig. S5). When proline 2 was included as a *cis* conformation in the CYANA calculations for isomer 1, the target function average for the granulin connectivity increased approximately 3.7-fold comparted to structures calculated with a *trans* conformation and showed one distance violation. This result supports the conclusion that proline 2 adopts the *trans* conformation.

**Figure 3:**
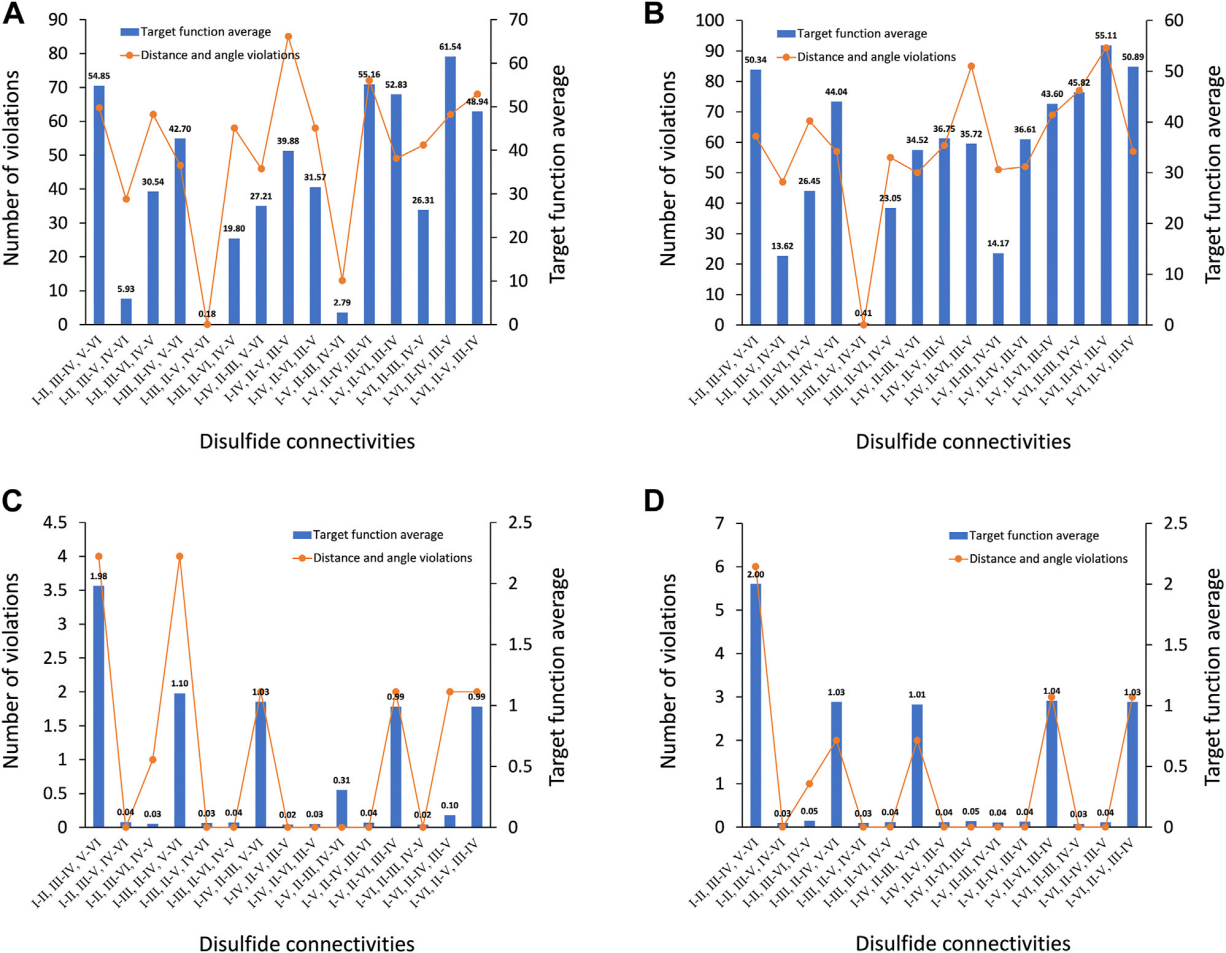
**Disulfide bonds connectivity analysis for GRN-P4A isomers.** Target function values derived from CYANA (31) calculations, based on NMR experimental data for the 15 possible disulfide connectivities of GRN-P4A isomers, are shown as *blue bars*. Distance and angle restraint violations are represented by an *orange line* with *circular markers*. The cut-off for angle restrains is 2.5°, the cut-off for distance bound violations is 0.2 Å. *A*, isomer 1 lowest target function corresponds to the granulin disulfide connectivity (CysI-CysIII, CysII-CysV, and CysIV-CysVI), with no violations. The majority of alternative disulfide connectivities display target function values exceeding the lowest by a factor of 15 or more, with violations ranging from 10 to 90. *B*, isomer 2 lowest target function is also associated with the granulin disulfide connectivity (Cys^I^-Cys^III^, Cys^II^-Cys^V^, and Cys^IV^-Cys^VI^), with no violations. Alternative disulfide connectivities show target function values exceeding the lowest by a factor of 30 or more, with violations ranging from 10 to 90. *C*, isomer 3 target functions are distributed across a range of approximately 0.04 to 0.02 for most of the connectivities (10 out of 15), with no violations. *D*, isomer 4 target functions are distributed across a range of approximately 0.05 to 0.03 for most of the connectivities (10 out of 15), with no violations.

The granulin disulfide connectivity pattern (Cys^I^-Cys^III^, Cys^II^-Cys^V^, and Cys^IV^-Cys^VI^) resulted in the lowest target function values of 0.18 for isomer 1 and of 0.41 for isomer 2 with no violations of distance or angle restraints in either isomer. The majority of the alternative disulfide combinations exhibited target function values exceeding the lowest by a factor of 15 or more, with violations ranging from 10 to 90, often involving restraints directly associated with the disulfide bridges. For isomers 3 and 4, a defined disulfide connectivity could not be assigned, as the target function values were distributed across a range of approximately 0.02 to 0.05 for most of the connectivities (approximately 10 out of 15 for each isomer), with no violations (Fig. 3, *A–D*). This result is consistent with the structural disorder predicted based on the chemical shift analysis.

**Figure 4:**
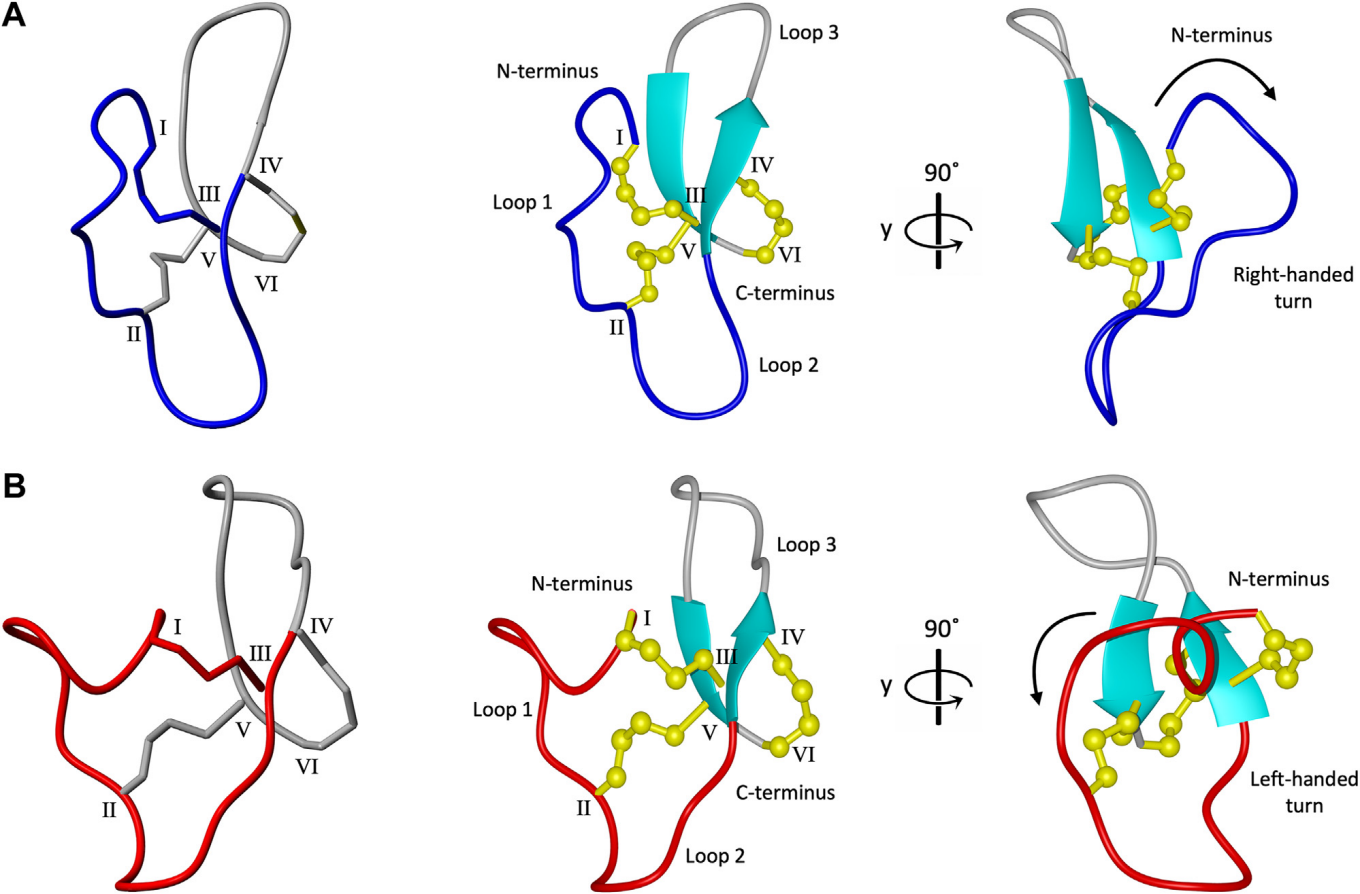
**GRN-P4A isomer one and two double lariat knot and lowest target function three-dimensional structure comparison**. The two interconnected lariats (*left*) are represented with different colors, *grey* and *blue* for isomer 1 (*A*) and *gray* and *red* for isomer 2 (*B*). The lowest target function structures of the two topological isomers were calculated using NMR spectroscopy. The main structural features (*middle*) and a 90° rotation along y-axis showing the orientation of Loop 1 turn (*right*) are reported for both isomers. The cysteine residues are denoted with Roman numerals (I-VI), while disulfide bonds are represented in *yellow* (with the four *yellow*-colored atom representing the β-methylene group (-CH_2_-) and the sulfur atom (-S-) of each cysteine residue involved in the formation of the disulfide bridge). β-sheets are shown in *cyan* for structural distinction.

GRN-P4A isomers 1 and 2 exhibit a mini-granulin fold consisting of a β-hairpin at the C-terminus, surrounded by three loops and turns (17). These structural elements are stabilized into a compact arrangement by disulfide bonds between cysteine residues, where cysteine 1 is connected to cysteine 14, 8 to 23 and 15 to 24. The primary conformational differences between the two isomers are localized to Loop 1 (cysteine 1 to cysteine 8) and Loop 2 (cysteine 8 to cysteine 14), with root mean square deviations (RMSD) of 2.5 Å and 1.8 Å respectively, when superposed. Structurally, these isomers form a highly knotted system characterized by two stacked lariat motifs. In this system, lariat one extends from threonine 13 to cysteine 1, with cysteine 15 forming the tail, while lariat two extends from glycine 22 to cysteine 24, with cysteine 8 serving as the tail. This structure can be described as a double-lariat knot (DLK), in which the two lariats are interconnected, and the tail of one lariat attaches to the loop of the other. Loop 1 adopts distinct conformations in isomers 1 and 2 that do not interconvert. In these forms, the Cα-Hα bond exhibit inverted geometries relative to the sidechain disulfide bond, with the backbone assuming a right-handed turn in isomer 1 and a left-handed turn in isomer 2 (Fig. 4, *A–B*). These findings suggest the two isomers, while displaying similar overall three-dimensional structures, represent a form of topological isomerism, likely driven by the conformational changes occurring in Loop 1 between Cys^1^ and Cys^8^. This structural variation may be attributed to restricted rotation around the proline 2 residue, which influences the distinct loop conformations and stabilizes each isomer in its unique form. A substantial number of cross-peaks were observed in the NOESY spectrum, indicating that both isomers adopt a well-defined structure. In contrast, isomers 3 and 4 are characterized by more dynamic and disordered structures, lacking β-sheet elements (Fig. 2*B*). The absence of well-defined secondary structures in these isomers likely contributes to their altered biological activities (Cell proliferation assay).

NMR spectra of isomer 1 were recorded at the three different temperatures of 290 K (standard conditions), 310 K (physiological temperature), and 283 K (lower temperature) to assess potential interconversion into isomer 2, the major oxidation product. Secondary shift analysis of GRN-P4A isomer 1 across these temperatures showed no significant variation (Fig. S6), indicating that no temperature-induced structural changes occur within this range. This finding suggests that isomer 1 maintains a stable three-dimensional conformation and is distinct from isomer 2, with no evidence of interconversion.

### AlphaFold structure prediction

To further examine the three-dimensional conformation of GRN-P4A, structural analysis was carried out using AlphaFold 3 (24). The five predicted models showed confidence scores (plDTT) ranging from 74 to 58, with higher scores indicating greater confidence in the predicted structure. All models shared the experimentally determined disulfide connectivity (Cys^I^-Cys^III^, Cys^II^-Cys^V^, and Cys^IV^-Cys^VI^), along with the presence of a β-hairpin secondary structure at the C-terminus. A 90° rotation along the y-axis revealed that all predicted structures exhibit a left-handed turn at Loop 1, consistent with the experimental findings for isomer 2 (Fig. S7).

In contrast, superposition of the cysteine residues from the highest-confidence AlphaFold model with the lowest target function structure calculated from the acquired NMR data of isomers 1 and 2 revealed greater similarity to isomer 1, with root mean square deviations of 1.1 Å for isomer 1 and 2.0 Å for isomer 2. A lower RMSD indicates that isomer 1 more closely matches the predicted AlphaFold structure. The two isomeric structures were also aligned with the AlphaFold predicted structure for the full-length sequence of *Ov*-GRN-1 (GenBank code ACJ83119.1), as the three-dimensional structure has not yet been experimentally determined. The alignment was based on the cysteine residues forming the native disulfide bonds (Cys^I^-Cys^III^ and Cys^II^-Cys^V^). Again, isomer 1 was more consistent with the AlphaFold predicted structure, with an RMSD three times lower than that of isomer 2 (data not shown). Further analysis of Loop 1, which is critical to topological isomerism, demonstrated that the AlphaFold-predicted structure aligns more closely with isomer 1 than isomer 2, with RMSD values of 1.0 Å and 2.3 Å, respectively. Additionally, the examination of Loop 2 located between residues 8 and 14, previously identified as crucial for bioactivity (25), revealed significant structural variability. When superposed to the highest-ranked AlphaFold model, the RMSD for Loop 2 in isomer 1 was 0.7 Å, whereas in isomer 2, it was notably higher at 1.3 Å.

### Role of proline 2 in the topological isomers

**Figure 5:**
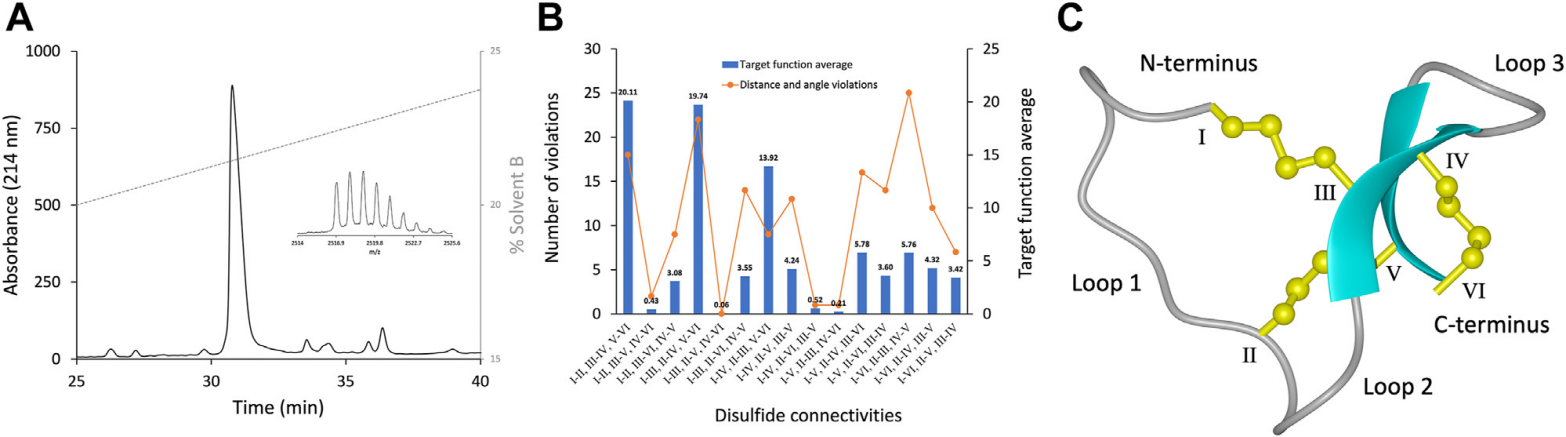
**GRN-L1m RP-HPLC post-oxidation chromatogramand structural analysis**. *A*, analytical RP-HPLC profile of fully oxidized GRN-L1m, displaying one sharp peak eluting at 30.78 min. Inset: MS spectrum of the compound [M + H]^+^ at *m/z* 2516.9 (theoretical [M + H]^+^ = 2517.9 *m/z*, error 397.2 ppm). *B*, disulfide bond connectivity analysis for GRN-L1m fifteen possible combinations. Target function values derived from CYANA (31) calculations, based on NMR experimental data for the 15 possible disulfide connectivities, are shown as *blue bars*. Distance and angle restraint violations are represented by an *orange line* with *circular markers*. The cutoff for angle restrains is 5°, the cut-off for distance bound violations is 0.2 Å. *C*, GRN-L1m (PDB code: 9MXG) three-dimensional structure with the disulfide bond connectivity corresponding to the lowest target function. The cysteine residues are labeled using Roman numerals (I-VI), the disulfide bonds are colored in *yellow* (with the four *yellow*-colored atom representing the β-methylene group (-CH_2_-) and the sulfur atom (-S-) of each cysteine residue involved in the formation of the disulfide bridge). β-sheets are shown in *cyan* for structural distinction. The model shows a mini-granulin fold with a granulin connectivity (Cys^I^-Cys^III^, Cys^II^-Cys^V^, and Cys^IV^-Cys^VI^).

GRN-Loop 1 mutant (GRN-L1m) was chemically synthesized using Fmoc solid-phase peptide synthesis. In this peptide, proline 2 was replaced with glycine to test the hypothesis that the conformational rigidity imposed by proline is responsible for the formation of isomers 1 and 2. Oxidation of GRN-L1m followed the same protocol as standard GRN-P4A, resulting in the generation of a single main product with no evidence of isomer formation. The RP-HPLC trace post-oxidation shows a distinct, sharp eluting peak at approximately 31 min, representing 95% of the oxidation products (Fig. 5*A*).

NMR analysis of GRN-L1m was conducted following the method previously described for GRN-P4A isomers (Structural analysis by NMR spectroscopy). This analysis confirmed the presence of a mini-granulin fold with the granulin disulfide connectivity (Cys^I^-Cys^III^, Cys^II^-Cys^V^, and Cys^IV^-Cys^VI^) (Fig. 5, *B*–*C*) and displayed a target function value of 5.83 × 10^-2^, with no significant distance or angle violations, supporting this configuration as the most probable. Alternative disulfide arrangements exhibited target function values at least 3.6 times higher, accompanied by 1 to 25 violations, primarily in restraints associated with disulfide bridges.

Comparative structural analysis through superposition of GRN-L1m with GRN-P4A isomers 1 and 2 revealed a closer alignment with isomer 1, consistent with findings from secondary αH shift analysis (Fig. S8). The main conformational differences were localized in Loop 1, with root mean square deviation values of 1.8 Å and 2.7 Å for superpositions of GRN-L1m with isomers 1 and 2, respectively. Superposition of cysteine residues and Loop 2 of GRN-L1m onto GRN-P4A isomers showed minimal differences, with RMSD values of 1.7 Å and 1.4 Å for cysteines, and 0.9 Å and 0.9 Å for Loop 2, when compared with isomers 1 and 2, respectively.

### Molecular dynamics simulations

**Figure 6:**
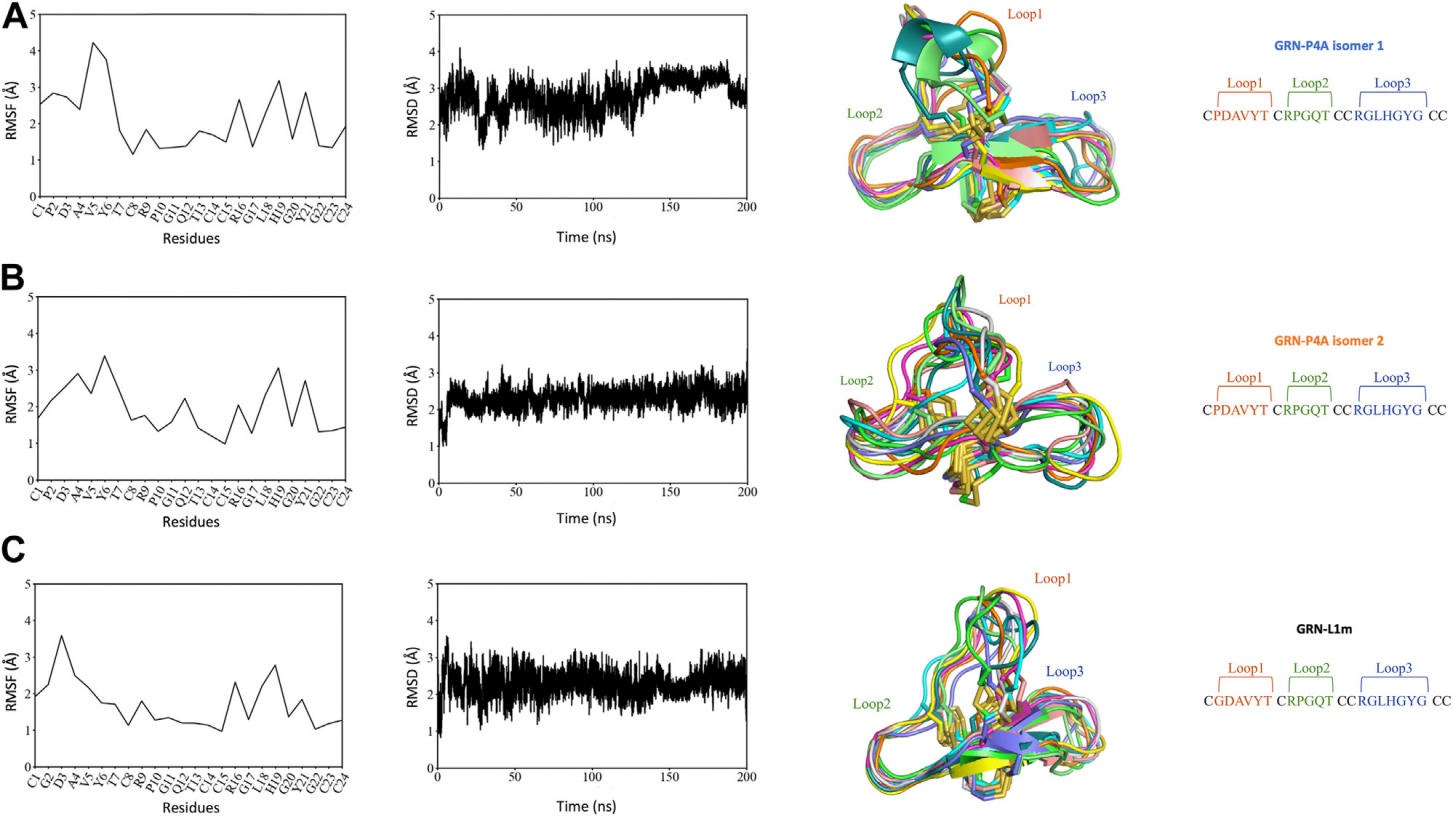
**Structural and dynamic analysis of GRN-P4A variants**. Data for GRN-P4A isomer 1 (*A*), GRN-P4A isomer 2 (*B*), and GRN-L1m (*C*) were obtained from 200 ns molecular dynamics simulations using the ff19SB force field (36) in AMBER20 (35). For each peptide, four analyses are presented from *left* to *right* (1): root mean square fluctuation (RMSF) over the 200 ns simulation period (2); root mean square deviation (RMSD) of backbone atoms over 200 ns, indicating structural stability (3); three-dimensional structural representation highlighting 10 conformations of three dynamic loops (Loop1, Loop2, Loop3), extracted at equal intervals throughout the simulation; and (4) amino acid sequence with annotated loop regions corresponding to those shown in the structural representation.

Molecular dynamics simulations of GRN-P4A isomer 1, GRN-P4A isomer 2, and GRN-L1m were performed to further investigate their structural stability and dynamic behavior. Root mean square fluctuation (RMSF) analysis (Fig. 6, *A*–*C*, first column) revealed distinct patterns of local flexibility across the three peptides. GRN-P4A isomers exhibited elevated fluctuations in Loop 1, indicative of increased mobility in this region. In contrast, Loop 2 showed minimal RMSF values, likely due to stabilization by its β-sheet secondary structure. Loop 3 demonstrated intermediate flexibility, with RMSF values falling between those of Loop 1 and Loop 2. Interestingly, despite containing a higher proportion of glycine residues, typically associated with increased flexibility, Loop 3 appeared less mobile than Loop 1. This may reflect the structural context in which the glycine residues are positioned, potentially constrained by local interactions that limit conformational freedom. Overall, GRN-P4A isomer 1 displayed the highest degree of mobility, particularly at the N-terminal region and Loop 3, whereas GRN-L1m exhibited reduced fluctuations across all loops, consistent with a more conformationally constrained architecture.

Backbone RMSD trajectories (Fig. 6, *A*–*C*, second column) show that all three peptides maintained overall structural stability throughout the 200 ns simulation. GRN-P4A isomer 1 exhibited slight deviations after 150 ns, suggesting late-stage conformational adjustments. In contrast, both isomer 2 and GRN-L1m showed more stable consistent RMSD profiles, with average values stabilizing between 2 and 3 Å.

The three-dimensional structural ensembles (Fig. 6, *A*–*C*, third column), derived from ten conformations sampled at regular intervals, further highlight the dynamic variability of the loop regions. GRN-P4A isomer 1 displayed pronounced conformational diversity in both Loop 1 and Loop 3, consistent with its higher RMSF values. GRN-P4A isomer 2 maintained relatively ordered loop structures, particularly in Loop 3. GRN-L1m exhibited a more restrained dynamic profile, with Loop 2 remaining structurally conserved across sampled frames.

### Cell proliferation assay

**Figure 7:**
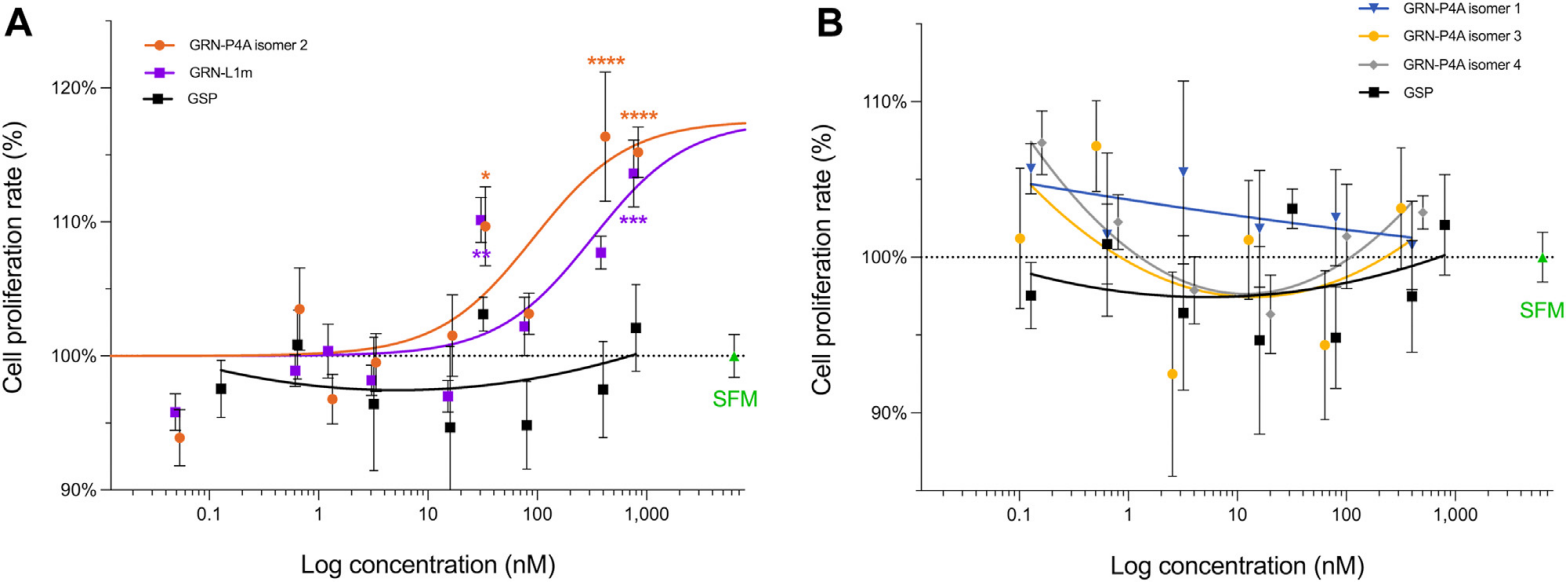
**Cell proliferation assay of GRN-P4A isomers and GRN-L1m on human hepatic stellate cells (LX-2)**. The five peptides were tested across a concentration range of 0.16 nM to 800 nM using the LX-2 human hepatic *stellate cell line*. Two negative controls were included: serum-free media (SFM) and a scrambled GRN-P4A peptide (GSP). The graphs display variable slope dose–response curves fitted to proliferation data measured 4 days after a single treatment application. *A*, GRN-P4A isomer 2 and GRN-L1m both induced cell proliferation, with isomer 2 exhibiting greater potency (EC_50_ = 92 nM; 95% CI) compared to GRN-L1m (EC_50_ = 318 nM; 95% CI). *B*, GRN-P4A isomers 1, 3, and 4 did not produce a statistically significant proliferative response at concentrations up to 400 nM. Proliferation is expressed relative to the scrambled peptide control (GSP) and presented as mean ± SEM. Statistical significance was assessed using one-way ANOVA against the peptide control. ∗ = *p* < 0.1, ∗∗ = *p* < 0.01, ∗∗∗ = *p* < 0.001, ∗∗∗∗ = *p* < 0.0001.

Real-time cell proliferation assays using xCELLigence technology were performed to assess the effects of GRN-P4A isomers and the mutant peptide GRN-L1m on the human hepatic stellate cell line LX-2. GRN-P4A isomer 2 showed the most potent proliferative effect, with an EC_50_ of 93 nM when compared to the negative control peptide GSP (scrambled version of GRN-P4A) (Fig. 7*A*). In contrast, isomers 1, 3, and 4 did not produce a statistically significant response up to concentrations of 400 nM (Fig. 7*B*). GRN-L1m also promoted LX-2 cell proliferation, although with lower potency than isomer 2, displaying an EC_50_ of 318 nM (Fig. 7*A*).

## Discussion

This study highlights the structural complexity and bioactivity of isomers in disulfide-rich peptides, with a focus on GRN-P4A. GRN-P4A is a mutant peptide derived from *Ov*-GRN12 − 35_3s, a truncated form of the liver fluke granulin *Ov*-GRN-1, known for its potent wound healing properties. Previous studies have demonstrated that the mutation of proline 4 in GRN-P4A leads to improved oxidative folding yields and enhanced wound healing activity in a mouse model (16), surpassing the efficacy of the FDA-approved treatment, Regranex (26), positioning it as a promising candidate for further development in wound healing therapies. However, further structural characterization has been limited by the presence of multiple conformations observed in NMR spectra thought to be caused by *cis/trans* isomerization of proline residues.

Our findings show that these additional NMR peaks are generated by the coexistence of two distinct topological isomers that share an identical disulfide connectivity but differ in their three-dimensional structures. The improved RP-HPLC separation method designed to purify GRN-P4A after oxidative folding allowed us to isolate these isomers into two distinct elution fractions. Isomer 2 displayed a broader and more intense peak compared to isomer 1, representing the major product of oxidative folding. NMR analysis revealed that both isomers share the same disulfide connectivity and a beta-hairpin structure at the C-terminus, but they differ in the first inter-cysteine loop around proline 2, with minor differences in Loop 2.

The substitution of proline 2 with glycine in a GRN-P4A mutant further supported the role of proline in governing topological isomerism. This mutation resulted in the formation of a single predominant product after oxidative folding, consistent with proline 2 being a key determinant of isomer differentiation. Unlike proline, which imposes rigidity and steric constraints due to its cyclic pyrrolidine ring, glycine confers greater conformational flexibility. This increased backbone flexibility appears to allow the peptide to adopt a single, thermodynamically stable conformation, thereby reducing the potential for multiple folding patterns and favoring one energetically preferred structure over several isomeric forms. These results demonstrate that the rigidity imposed by proline 2 is essential for maintaining structural divergence between isomers, and that targeted amino acid substitutions can be strategically used to modulate conformational heterogeneity in disulfide-rich peptides.

Interestingly, despite varying temperature conditions, the isomers remained stable and did not interconvert. Isomer 1 retained its structure across all tested temperatures (283K, 290K, and 310K), indicating that both topological isomers adopt well-defined and energetically stable conformations. While proline residues have been observed to direct peptide folding pathways in other systems (27), our findings add new insights into oxidative folding by revealing that stable, non-interconverting isomers can arise from subtle differences in loop flexibility.

From a biological perspective, the observed differences in cell proliferation activity between the isomers are particularly significant. Isomer 2, the major oxidation product, exhibited the strongest proliferation effect, suggesting that its unique structural features may enhance interactions with cellular targets involved in growth regulation. In contrast, although structurally similar to isomer 2, isomer 1 demonstrated a weaker bioactivity profile, highlighting how minor conformational changes can significantly influence biological function. These findings align with a previous study where the three loops of *Ov*-GRN12 − 35_3s were synthesized as individual peptides and tested for bioactivity. Notably, Loop 2 was identified as the most active for *in vitro* cell proliferation (25), and the slight conformational differences in this loop between the two isomers may explain their varying bioactivities. Further studies are required to elucidate the biological target(s) of these isomers and determine properties such as cell penetration.

The MD simulation results reveal notable differences in the dynamic behavior of GRN-P4A isomer 1, GRN-P4A isomer 2, and GRN-L1m, with implications for their structural stability and potential functional roles. Interestingly, GRN-P4A isomer 1 exhibited greater conformational flexibility, particularly in Loop 1 and Loop 3, as indicated by elevated RMSF values and pronounced dispersion of these regions within the three dimensional-structural ensembles. Notably, this variant also demonstrates more modest biological activity compared to isomer 2, suggesting a possible correlation between loop flexibility and functional efficacy.

Moreover, the distinct rotational orientation of Loop 1, right-handed in isomer one and left-handed in isomer 2, likely contributes to the differences in bioactivity, a phenomenon previously observed with topological isomers of heat-stable enterotoxin (STa) (15). The additional oxidation isomers exhibited distinct behaviors as well. Isomer 4, which adopts a less ordered structure, promoted cell proliferation at higher concentrations, while isomer 3 displayed cytotoxicity, likely due to peptide aggregation. These results underscore the importance of thoroughly analyzing individual isomers in therapeutic applications, as even minor structural variations can result in markedly different biological outcomes.

The failure of AlphaFold to predict the structural distinctions of Loop 1, despite accurately identifying the disulfide bond connectivity, further underscores the challenge of modeling topological isomerism in disulfide-rich peptides. These findings suggest that while predictive tools like AlphaFold are valuable, they may not fully capture the conformational intricacies that arise during oxidative folding. This limitation highlights the necessity of experimental validation, especially when subtle conformational differences can profoundly influence biological function.

This study provides important implications for the development of GRN-P4A as a therapeutic agent. The ability to selectively generate and control topological isomers during peptide synthesis and folding could significantly impact both the efficacy and the safety of disulfide-rich peptides in clinical applications. Given the potential of GRN-P4A in wound healing, understanding the contribution of individual isomers to its bioactivity will be essential for optimizing its therapeutic potential.

In conclusion, this study provides novel insights into the oxidative folding mechanisms of disulfide-rich peptides, emphasizing the critical role of topological isomerism in modulating both structure and function. The differences in bioactivity between the two main isomers, (isomers 1 and 2) indicate that appropriate gradients are required in the purification process to optimize the purity of the bioactive form. These findings will aid in refining drug development strategies, ensuring enhanced pharmacokinetic properties and greater scalability in clinical applications. These findings not only deepen our understanding of peptides such as GRN-P4A but also open new avenues for the rational design of peptide-based therapeutics.

## Experimental procedures

### Peptide synthesis and purification

Linear reduced GRN-P4A (CPDAVYTCRPGQTCCRGLHGYGCC) was synthesized by GenScript Biotech Corporation with a grade of purity ≥95% following previously described methods (16). The modified peptide, GRN-L1m (CGDAVYTCRPGQTCCRGLHGYGCC), where proline 2 has been changed for a glycine, was synthesized manually using solid-phase peptide synthesis with Fmoc chemistry. The assembly of the peptide was performed on 2-chlorotrityl chloride resin (PEPTIDES INTERNATIONAL). Amino acid derivates (Novabiochem) were activated using O-(1H-6- chlorobenzotriazol-1-yl)-1,1,3,3-tetramethyluronium hexafluorophosphate (HCTU) in peptide synthesis grade dimethylformamide (DMF). Deprotection of the Fmoc groups was achieved using 20% piperidine in DMF. Peptide was cleaved by treating it with a solution of 95% trifluoroacetic acid (TFA), 2.5% water and 2.5% triisopropyl silane (TIPS) for 2.5 h at room temperature. TFA was subsequently removed through nitrogen evaporation, followed by precipitation of the peptide using ice-cold diethyl ether. After filtration to remove excess ether, the peptides were dissolved in a 45% acetonitrile-water solution containing 0.1% TFA and then lyophilized. The resulting crude peptide was purified by RP-HPLC on an Agilent 1260 Infinity system equipped with a C_18_ preparative column (Phenomenex Jupiter 10 μm, C_18_, 300 Å pore size, 250 mm × 21.2 mm; Phenomenex), with a flow rate of 5 ml/min, and 1% gradient (0–60% B) of solvent B (90% acetonitrile/10% water/0.045% TFA (v/v/v)) in solvent A (water/0.05% TFA (v/v)).

Eluent detection was performed at 214 and 280 nm, and mass analysis was carried out using a 5800 MALDI TOF/TOF mass spectrometer (SCIEX, Framingham, MA, USA). For sample preparation, 0.75 μl of peptide solution was mixed with 0.75 μl of α-cyano-4-hydroxycinnamic acid (CHCA) matrix (Sigma-Aldrich) at a concentration of 7.5 mg/ml in 50% acetonitrile containing 0.1% TFA. The prepared mixture was then spotted onto a 384-well stainless-steel target plate. Prior to data acquisition, calibration was performed using the SCIEX TOF/TOF calibration mixture (PN#: 4,333,604; SCIEX). Spectra were acquired in linear and reflector positive ion mode from *m/z* 1000 to 10,000 and *m/z* 800 to 4500 and averaged over 2500 and 2000 laser shots, respectively.

### Peptide oxidation and purification

Disulfide bonds were formed by air oxidation of 0.2 mg/ml peptide in 100 mM ammonium bicarbonate (pH 8.5) on a stirring plate at room temperature for 48 h. The solution was filtered and monitored using RP-HPLC on a C_18_ analytical column (Kinetex 3.5 μm XB-C18 100 Å pore size, 150 × 4.6 mm; Phenomenex) and purified on a C_18_ semi-preparative column (Aeris PEPTIDE 5 μm, XB-C18, 100 Å pore size, 250 × 10.0 mm; Phenomenex), with a 0.25% gradient (15–30% solvent B) and a flow rate of 1 and 3 ml/min respectively. The purity of oxidized peptides was assessed using RP-HPLC on a C_18_ analytical column (Aeris PEPTIDE 3.5 μm, XB-C18, 100 Å pore size, 150 × 2.10 mm; Phenomenex), peaks were detected at 214 nm with a gradient of 0 − 30% solvent B in 60 min at 0.25 ml/min. Peptide MS and MS/MS analysis were performed using MALDI TOF/TOF mass spectrometry. MS/MS spectra were acquired using a precursor *m/z* of 2556.72 averaged over 2500 laser shots.

### NMR spectroscopy and structure determination

Lyophilized peptides were rehydrated in a mixture of 500 μl water and 50 μl D_2_O, to obtain a final concentration of 0.2 mM. NMR spectra were recorded at temperatures of 283, 290 and 310 K using a 600 MHz Bruker Avance III spectrometer (Bruker) equipped with a cryoprobe. Chemical shifts were referenced to external DSS (sodium trimethylsilylpropanesulfonate). Two-dimensional spectra included [^1^H,^1^H]-TOCSY, [^1^H,^1^H]-H/D exchange TOCSY, [^1^H,^1^H]-NOESY, [^1^H,^1^H]-COSY, [^1^H,^1^H]-exclusive correlation spectroscopy, [^1^H,^13^ C]-HSQC, and [^1^H,^15^ N]-HSQC with TOCSY and NOESY mixing times of 80 ms and 200 ms, respectively. NMR data processing and analysis were performed using TopSpin (Bruker), and peak assignments were made using CcpNMR Analysis (Collaborative Computing Project for NMR) based on the method outlined by Wuthrich (28, 29). Backbone torsion-angle restraints for phi and psi were predicted from ^1^H and ^13^C chemical-shift analyses, were predicted using TALOS-N (30). The disulfide bond connectivity for all theoretically possible conformations (with six cysteines generating 15 possible disulfide connectivities) was included in the structure calculations. Distance restraints were obtained from the automated assignment of [^1^H,^1^H]-NOESY spectra, and the 20 structures with the lowest energy were generated using the CYANA program (31). The three-dimensional structures were visualized using MOLMOL (32). The αH secondary shifts were determined by subtracting random coil ^1^H NMR chemical shifts (19) from the experimental αH chemical shifts. The structural data have been deposited in the Protein Data Bank (PDB) (33) under the codes 9MXF, 9MXE, and 9MXG, corresponding to GRN-P4A isomer 1, GRN-P4A isomer 2, and GRN-L1m, respectively. The associated chemical shift data have been submitted to the Biological Magnetic Resonance Bank (http://www.bmrb.wisc.edu/) (34) under the BRMB IDs 31,226, 31,225, and 31,227 for GRN-P4A isomer 1, GRN-P4A isomer 2, and GRN-L1m, respectively.

### Molecular dynamics simulations

Molecular dynamics (MD) simulations of GRN-P4A peptides (isomer 1, isomer 2, and L1m) were conducted using AMBER20 (35) with the ff19SB force field (36) for protein parameterization. The peptides were solvated in an OPC water box with a 10 Å buffer, and the electrical charge was neutralized using Na^+^ ions. System construction and setup were carried out using xLEaP in AMBER20 (35) and energy minimization was performed before the MD simulations.

Energy minimization was performed in two stages. The first stage involved a restrained minimization using 3000 steps of the steepest descent method followed by 3000 steps of the conjugate gradient method, with the solute restrained with a force constant of 10 kcal mol^-1^ Å^-2^. This was followed by a second, unrestrained minimization. The system was then equilibrated by gradually heating from 50 K to 300 K over 100 ps under constant volume and temperature conditions (NVT), with solute restrained with a harmonic force of 5 kcal mol^-1^ Å^-2^.

Production MD simulations were then performed under constant number of particles, pressure and temperature conditions without restraints. Throughout the simulation, structural stability was monitored by calculating the root mean square deviation (RMSD) of the peptide backbone.

### Cell culture

The LX-2 human *hepatic stellate cell* line was generously donated by Dr Lionel Hebbard. Cells were cultured and maintained in Dulbecco’s Modified Eagle Medium/Nutrient Mixture F-12 (DMEM/F12) with GlutaMAX (Gibco) with 2 × antibiotic/antimycotic solution (penicillin, streptomycin, and amphotericin B), supplemented with 2% fetal bovine serum (FBS) (Gibco), at 37°C and 5% CO_2_. For the negative control, cells were treated with scrambled granulin peptide (GSP) or serum free media diluent. Dried peptide powder was resuspended in Milli-Q water to 170 μM and diluted in serum-free DMEM/F12 media.

### Cell proliferation monitoring in real time using xCELLigence

Cell proliferation was monitored in real time using the xCELLigence SP system (ACEA Biosciences), following previously established method (37). LX2 cells were seeded into a 96-well xCELLigence E-plate (Agilent) at a density of 3000 cells per well in 150 μl of medium, and placed in the xCELLigence system at 37°C with 5% CO_2_, for overnight monitoring (38). Treatments were applied in serum-free medium at 20 μl per well, achieving final concentrations of 0.64 nM to 800 nM, with six replicates per treatment. The cell index (CI) was continuously monitored using the Real-Time Cell Analysis (RTCA) software (Agilent), with measurements taken every hour over 6 days following treatment. Cell indices were normalized to the baseline values prior to treatment, and cell proliferation was expressed as the relative number of cells compared to the negative control GRN-scrambled peptide (GSP). Statistical analysis was conducted using two-way ANOVA test with Holm-Sidak’s correction comparing controls to treatments at each timepoint with GraphPad Prism 9.3.1 (www.graphpad.com).

## Data availability

Data is presented in the manuscript.

## Supporting information

This article contains supporting information (19, 24, 39).

## Conflict of interest

The authors declare that they have no conflicts of interest with the contents of this article.
