# Supplementary material for: Topological isomers of a potent wound healing peptide: Structural insights and implications for bioactivity
Source: J Biol Chem. 2025 Jun 4;301(7):110340. doi: 10.1016/j.jbc.2025.110340 (PMC12270677; doi:10.1016/j.jbc.2025.110340)
Supplement: Supplementary Material [file mmc1.docx]

**Topological isomers of a potent wound healing peptide: structural insights and implications for bioactivity**

**Tiziano Raffaelli^1^, David T. Wilson^1^, Mehdi Mobli^2^, Michael J. Smout^1^, Guangzu Zhao^1^, Rozita Takjoo^1^, **Paramjit S. Bansal^1^**, Alex Loukas^1^, Rilei Yu^3, 4^, Zixuan Zhang^3, 4^ and Norelle L. Daly^1*^**

^1^Australian Institute of Tropical Health and Medicine, James Cook University, Cairns, Australia; ^2^Centre for Advanced Imaging, The University of Queensland, Queensland, Qld, Australia; ^3^Key Laboratory of Marine Drugs, Chinese Ministry of Education, School of Medicine and Pharmacy, Ocean University of China, Qingdao, 266003, China; ^4^Laboratory for Marine Drugs and Bioproducts, Qingdao Marine Science and Technology Center, Qingdao, 266237, China

^∗^Correspondence should be addressed to: Norelle L. Daly, Australian Institute of Tropical Health and Medicine, James Cook University, QLD 4878, Australia. Tel.: +61 [(07) 4232 1815](mailto:(07)%204232%201815), E-mail: [norelle.daly@jcu.edu.au](mailto:norelle.daly@jcu.edu.au)

Supporting Figures

**
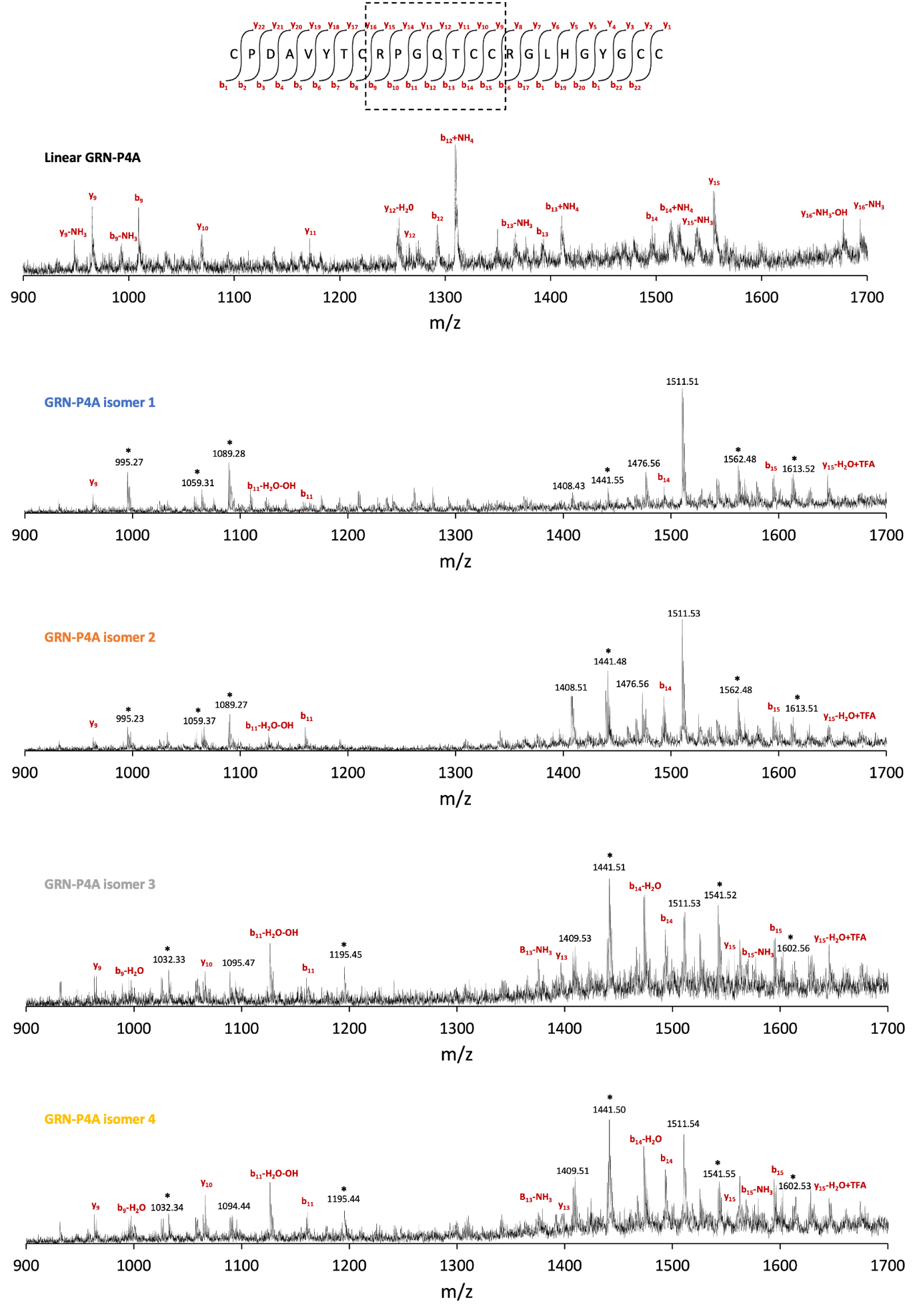
**

| **Peptide Variant** | **Fragment Ion (b/y/internal)** | **Sequence** | **Theoretical m/z** | **Measured m/z** | **Mass Error (ppm)** | **Notes** |
| --- | --- | --- | --- | --- | --- | --- |
| Linear | y_9_-NH_3_ | CRGLHGYGCC | 948.38 | 948.31 | -73.81 | Adduct |
|  | y_9_ | CRGLHGYGCC | 965.41 | 965.30 | -113.94 | — |
|  | b_9_-NH_3_ | CPDAVYTCR | 992.40 | 992.42 | +20.15 | — |
|  | b_9_ | CPDAVYTCR | 1009.42 | 1009.32 | -99.07 | — |
|  | y_10_ | CCRGLHGYGCC | 1068.42 | 1068.30 | -112.32 | — |
|  | y_11_ | TCCRGLHGYGCC | 1171.43 | 1171.39 | -34.15 | — |
|  | y_12_-H_2_O | QTCCRGLHGYGCC | 1254.46 | 1254.33 | -103.63 | Adduct |
|  | y_12_ | QTCCRGLHGYGCC | 1272.47 | 1272.35 | -94.30 | — |
|  | b_12_ | CPDAVYTCRPGQ | 1292.56 | 1292.45 | -85.10 | — |
|  | b_12_+NH_4_ | CPDAVYTCRPGQ | 1310.59 | 1310.44 | -114.45 | Adduct |
|  | b_13_-NH_3_ | CPDAVYTCRPGQT | 1375.58 | 1375.41 | -123.58 | Adduct |
|  | b_13_ | CPDAVYTCRPGQT | 1392.60 | 1392.42 | -129.25 | — |
|  | b_13_+NH_4_ | CPDAVYTCRPGQT | 1410.63 | 1410.53 | -70.89 | Adduct |
|  | y_15_-NH_3_ | RPGQTCCRGLHGYGCC | 1537.58 | 1537.45 | -84.55 | Adduct |
|  | y_15_ | RPGQTCCRGLHGYGCC | 1554.61 | 1554.56 | -32.16 | — |
|  | y_16_-NH_3_-OH | CRPGQTCCRGLHGYGCC | 1676.68 | 1676.57 | -65.61 | Adduct |
|  | y_16_-NH_3_ | CRPGQTCCRGLHGYGCC | 1693.68 | 1693.52 | -94.47 | Adduct |
|  | | | | | | |
| Isomer 1 | y_9_ | CRGLHGYGCC | 963.39 | 963.30 | -93.42 | — |
|  | Internal Frag. | RPGQTCCR-H_2_O+TFA | 995.37 | 995.27 | -100.47 | Adduct, * |
|  | Internal Frag. | PDAVYTCRPG | 1059.41 | 1059.31 | -94.39 | * |
|  | Internal Frag. | VYTCRPGQTC-H_2_O | 1089.46 | 1089.28 | -165.22 | Adduct, * |
|  | b_11_-H_2_O-OH | CPDAVYTCRPG-H2O -OH | 1126.47 | 1126.32 | -133.16 | — |
|  | b_11_ | CPDAVYTCRPG | 1161.48 | 1161.43 | -43.05 | — |
|  | Internal Frag. | YTCRPGQTCCRGL-CO | 1408.62 | 1408.43 | -134.88 | Adduct |
|  | Internal Frag. | PGQTCCRGLHGYG+TFA | 1441.55 | 1441.55 | 0.00 | Adduct, * |
|  | Internal Frag. | AVYTCRPGQTCCRG-NH_3_ | 1476.61 | 1476.56 | -33.86 | Adduct |
|  | b_14_ | CPDAVYTCRPGQTC | 1492.59 | 1492.56 | -20.10 | — |
|  | Internal Frag. | CRPGQTCCRGLHGY | 1511.63 | 1511.51 | -79.38 | — |
|  | Internal Frag. | TCRPGQTCCRGLHG-H_2_O+TFA | 1562.60 | 1562.48 | -76.80 | Adduct,* |
|  | b_15_ | CPDAVYTCRPGQTCC | 1594.59 | 1594.49 | -62.71 | — |
|  | Internal Frag. | TCRPGQTCCRGLHGY-NH_3_ | 1613.66 | 1613.52 | -86.76 | Adduct, * |
|  | y_15_-H_2_O+TFA | RPGQTCCRGLHGYGCC+TFA | 1645.56 | 1645.49 | -42.54 | Adduct |
|  | | | | | | |
| Isomer 2 | y_9_ | CRGLHGYGCC | 963.39 | 963.40 | +10.38 | — |
|  | Internal Frag. | RPGQTCCR-H_2_O+TFA | 995.37 | 995.23 | -140.65 | Adduct, * |
|  | Internal Frag. | PDAVYTCRPG | 1059.41 | 1059.37 | -37.76 | * |
|  | Internal Frag. | VYTCRPGQTC-H_2_O | 1089.46 | 1089.27 | -174.40 | Adduct, * |
|  | b_11_-H_2_O-OH | CPDAVYTCRPG | 1126.47 | 1126.38 | -79.90 | — |
|  | b_11_ | CPDAVYTCRPG | 1161.48 | 1161.31 | -146.36 | — |
|  | Internal Frag. | YTCRPGQTCCRGL-CO | 1408.62 | 1408.51 | -78.09 | Adduct |
|  | Internal Frag. | PGQTCCRGLHGYG+TFA | 1441.55 | 1441.48 | -48.56 | Adduct, * |
|  | Internal Frag. | AVYTCRPGQTCCRG-NH_3_ | 1476.61 | 1476.56 | -33.86 | Adduct |
|  | b_14_ | CPDAVYTCRPGQTC | 1492.59 | 1492.52 | -46.90 | — |
|  | Internal Frag. | CRPGQTCCRGLHGY | 1511.63 | 1511.53 | -66.15 | — |
|  | Internal Frag. | TCRPGQTCCRGLHG-H_2_O+TFA | 1562.60 | 1562.48 | -76.80 | Adduct, * |
|  | b_15_ | CPDAVYTCRPGQTCC | 1594.59 | 1594.52 | -43.90 | — |
|  | Internal Frag. | TCRPGQTCCRGLHGY-NH_3_ | 1613.66 | 1613.51 | -92.96 | Adduct, * |
|  | y_15_-H_2_O+TFA | RPGQTCCRGLHGYGCC+TFA | 1645.56 | 1645.48 | -48.62 | Adduct |
|  | | | | | | |
| Isomer 3 | y_9_ | CRGLHGYGCC | 963.39 | 963.29 | -103.80 | — |
|  | b_9_-H_2_O | CPDAVYTCR | 989.40 | 989.32 | -80.86 | Adduct |
|  | Internal Frag. | TCRPGQTCC-CO+TFA | 1032.33 | 1032.33 | 0.00 | Adduct, * |
|  | y_10_ | CCRGLHGYGCC | 1065.39 | 1065.36 | -28.16 | — |
|  | Internal Frag. | AVYTCRPGQT+NH_4_ | 1094.54 | 1094.47 | -63.95 | Adduct |
|  | b_11_-H_2_O-OH | CPDAVYTCRPG | 1126.44 | 1126.40 | -35.51 | Adduct |
|  | b_11_ | CPDAVYTCRPG | 1161.48 | 1161.34 | -120.54 | — |
|  | Internal Frag. | YTCRPGQTCC-CO+TFA | 1195.41 | 1195.45 | +33.46 | Adduct, * |
|  | b_13_-NH_3_ | CPDAVYTCRPGQT | 1373.56 | 1373.52 | -29.12 | Adduct |
|  | y_13_ | GQTCCRGLHGYGCC | 1396.50 | 1396.51 | +7.16 | — |
|  | Internal Frag. | RPGQTCCRGLHGY-H_2_O | 1409.63 | 1409.53 | -70.94 | Adduct |
|  | Internal Frag. | PGQTCCRGLHGYG+TFA | 1441.55 | 1441.51 | -27.75 | Adduct, * |
|  | b_14_-H_2_O | CPDAVYTCRPGQTC | 1474.58 | 1474.49 | -61.03 | Adduct |
|  | b_14_ | CPDAVYTCRPGQTC | 1492.59 | 1492.53 | -40.20 | — |
|  | Internal Frag. | CRPGQTCCRGLHGY-H_2_O | 1511.63 | 1511.53 | -66.15 | Adduct |
|  | Internal Frag. | DAVYTCRPGQTCCR-CO+NH_4_ | 1541.67 | 1541.52 | -97.30 | Adduct |
|  | y_15_ | RPGQTCCRGLHGYGCC | 1550.57 | 1550.54 | -19.35 | — |
|  | b_15_-NH_3_ | CPDAVYTCRPGQTCC | 1577.56 | 1577.51 | -31.69 | — |
|  | b_15_ | CPDAVYTCRPGQTCC | 1594.59 | 1594.50 | -56.44 | Adduct |
|  | Internal Frag. | TCRPGQTCCRGLHGY-CO | 1602.69 | 1602.56 | -81.11 | Adduct, * |
|  | y_15_-H_2_O+TFA | RPGQTCCRGLHGYGCC | 1645.56 | 1645.46 | -60.77 | Adduct |
|  | | | | | | |
| Isomer 4 | y_9_ | CRGLHGYGCC | 963.39 | 963.32 | -72.66 | — |
|  | b_9_-H_2_O | CPDAVYTCR | 989.40 | 989.35 | -50.54 | Adduct |
|  | Internal Frag. | TCRPGQTCC-CO+TFA | 1032.33 | 1032.34 | +9.69 | Adduct, * |
|  | y_10_ | CCRGLHGYGCC | 1065.39 | 1065.32 | -65.70 | — |
|  | Internal Frag. | AVYTCRPGQT+NH_4_ | 1094.54 | 1094.44 | -91.36 | Adduct |
|  | b_11_-H_2_O-OH | CPDAVYTCRPG | 1126.44 | 1126.41 | -26.63 | Adduct |
|  | b_11_ | CPDAVYTCRPG | 1161.48 | 1161.36 | -103.32 | — |
|  | Internal Frag. | YTCRPGQTCC-CO+TFA | 1195.41 | 1195.44 | +25.10 | Adduct, * |
|  | b_13_-NH_3_ | CPDAVYTCRPGQT | 1373.56 | 1373.51 | -36.40 | Adduct |
|  | y_13_ | GQTCCRGLHGYGCC | 1396.50 | 1396.51 | +7.16 | — |
|  | Internal Frag. | RPGQTCCRGLHGY-H_2_O | 1409.63 | 1409.51 | -85.13 | Adduct |
|  | Internal Frag. | PGQTCCRGLHGYG+TFA | 1441.55 | 1441.50 | -34.68 | Adduct, * |
|  | b_14_-H_2_O | CPDAVYTCRPGQTC | 1474.58 | 1474.49 | -61.03 | Adduct |
|  | b_14_ | CPDAVYTCRPGQTC | 1492.59 | 1492.50 | -60.30 | — |
|  | Internal Frag. | CRPGQTCCRGLHGY-H_2_O | 1511.63 | 1511.54 | -59.54 | Adduct |
|  | Internal Frag. | DAVYTCRPGQTCCR-CO+NH_4_ | 1541.67 | 1541.55 | -77.84 | Adduct |
|  | y_15_ | RPGQTCCRGLHGYGCC | 1550.57 | 1550.54 | -19.35 | — |
|  | b_15_-NH_3_ | CPDAVYTCRPGQTCC | 1577.56 | 1577.55 | -6.34 | — |
|  | b_15_ | CPDAVYTCRPGQTCC | 1594.59 | 1594.50 | -56.44 | Adduct |
|  | Internal Frag. | TCRPGQTCCRGLHGY-CO | 1602.69 | 1602.53 | -99.83 | Adduct, * |
|  | y_15_-H_2_O+TFA | RPGQTCCRGLHGYGCC | 1645.56 | 1645.56 | 0.00 | Adduct |

**Figure S1. MALDI MS/MS fragmentation traces of linear GRN-P4A and oxidised isomers.** Samples were spotted on 384-well stainless-steel target plates using 0.75 μL of sample and 0.75 μL of α-cyano-4-hydroxycinnamic acid (CHCA; Sigma-Aldrich) matrix at 7.5 mg/mL in 50% MeCN/0.1% TFA. MS/MS spectra were acquired using a precursor *m/z* of 2556.72. The analysis generated comparable fragmentation patterns for isomers 1 and 2, as well as for isomers 3 and 4, suggesting potential structural similarities between these pairs of peptides. Notably, MS/MS analysis of the linear GRN-P4A peptide revealed the presence of the majority of expected b- and y-ions within the m/z range 900-1700 (selected due to its clear peak distribution), with no internal fragments detected. The identified b- and y-ions are annotated in the spectrum, while the masses of ambiguous peaks, potentially corresponding to internal fragments or adducts, are marked with an asterisk (*). A table listing the most likely ion assignments is provided below the spectra.


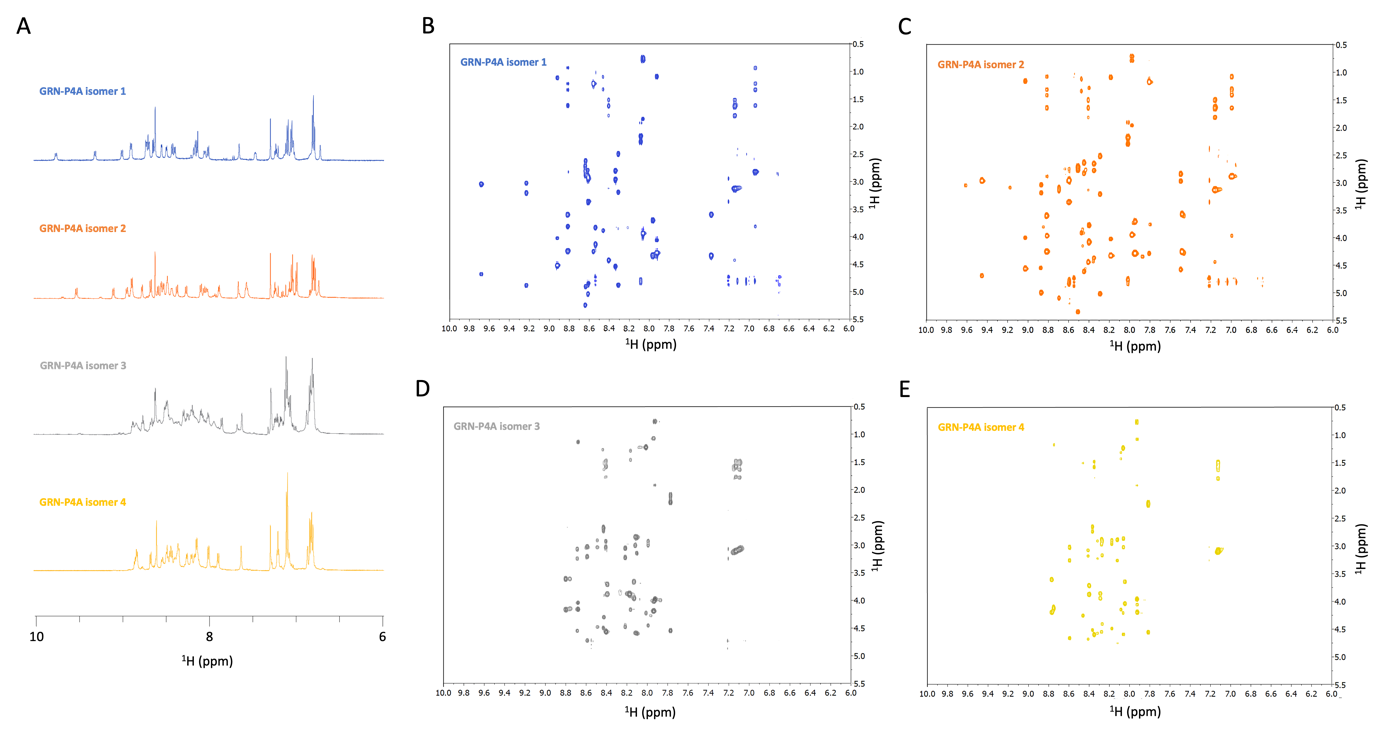


**Figure S2. Comparison of one-dimensional proton and [^1^H,^1^H]-TOCSY NMR spectra of the 4 GRN-P4A isomers.** Samples were dissolved in a solution of 500 μL water and 50 μL D_2_O, for a final peptide concentration of 0.2 mM. The experiments were carried out at 290 K using a 600 MHz Bruker Avance III spectrometer (see Experimental procedures). **(A)** One-dimensional proton spectra of the amide region (6-10 ppm) for the 4 isomers. The [^1^H,^1^H]-TOCSY NMR spectra of **(B)** isomer 1 and **(C)** isomer 2 show a good dispersion in the amide region, whereas **(D)** isomer 3 and **(E)** isomer 4 has significant overlap of the amide proton peaks.


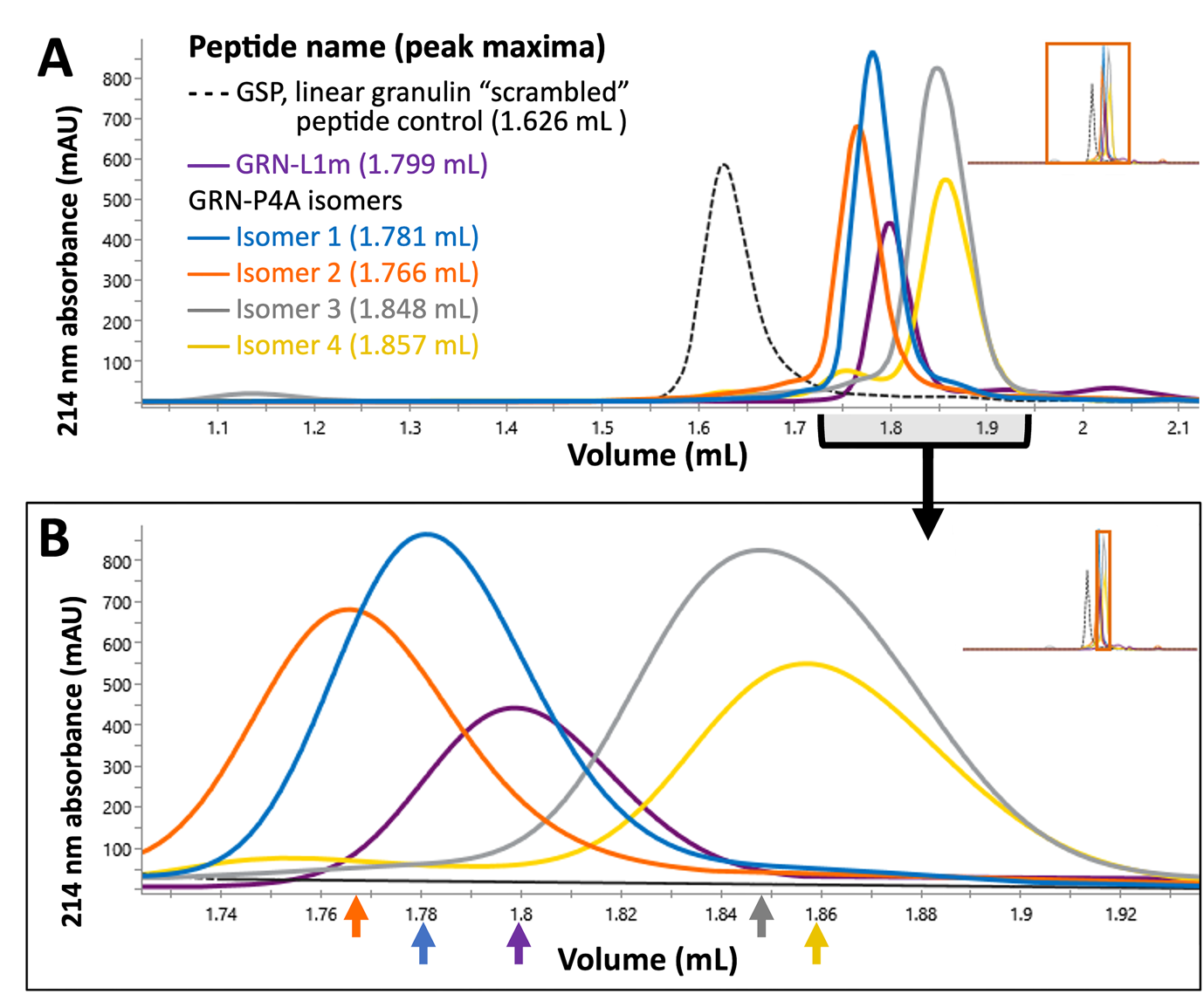


****Figure S3.** Fast protein liquid chromatography (FPLC) size exclusion peptide separation for GRNA-P4A isomers and GRN-L1m.** Under biological conditions (PBS pH 7.4) 10 µL of 170 µM peptide was separated at 0.02 mL/min at 10^o^C on a Superdex 30 increase 3.2/300 column (column volume ~2.1 mL) with an AKTA pure system. GSP, linear granulin “scrambled” peptide control is a peptide with the same amino acid composition as the GRN-P4A peptide but with a scramble sequence order and all cysteines replaced with alanine. *A*, Peptide peaks separation with 214nm absorbance plotted against elution volume from 1.0-2.1 mL. Inset shows the full separation trace. Bracket and arrow show magnified region in lower panel. *B,* Magnified section of panel A, highlighting the major peaks of interest. Colored arrows represent peak migration maxima for each peptide.


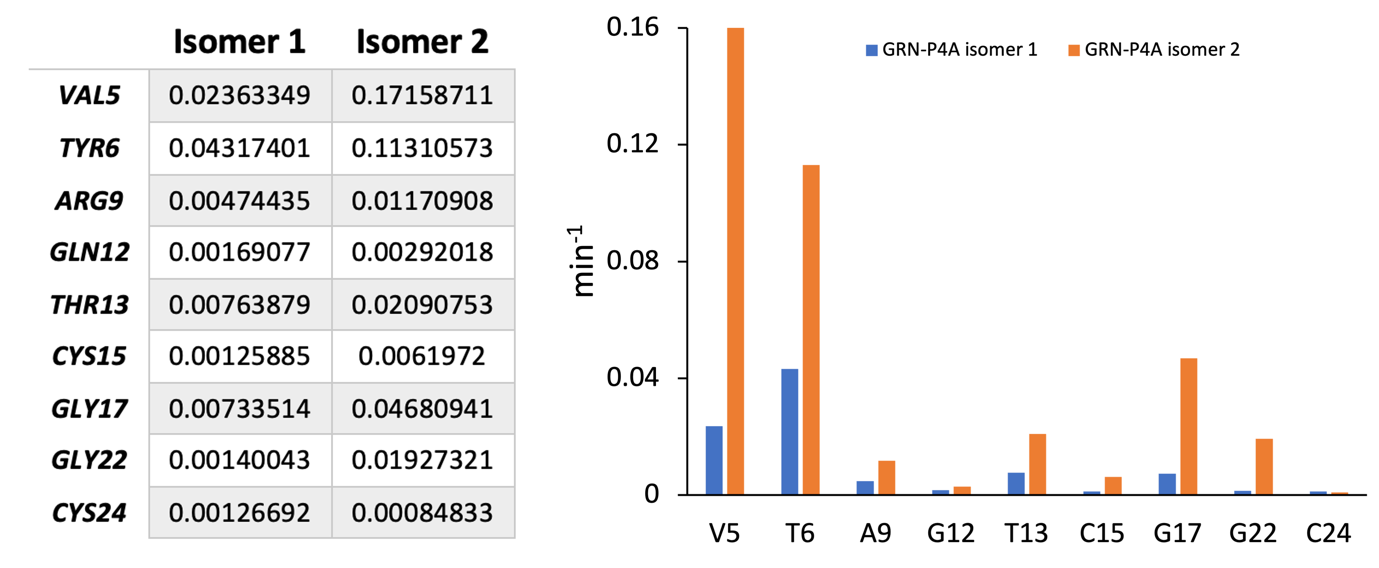


**Figure S4. Comparison of [^1^H,^1^H]-TOCSY slow exchange rates for GRN-P4A isomers 1 and 2.** Samples were dissolved in 500 μL of D_2_O, for a final peptide concentration of 0.8 mM. The experiments were carried out at 290 K using a 600 MHz Bruker Avance III spectrometer and spectra were recorded at intervals of 27 minutes. Exchange rates for amide protons were calculated by fitting the volume of the decaying signals over time to the equation *I* = *I_o_* exp(-*k*_ex_ x *t*) + *I* (*∞*) (1).


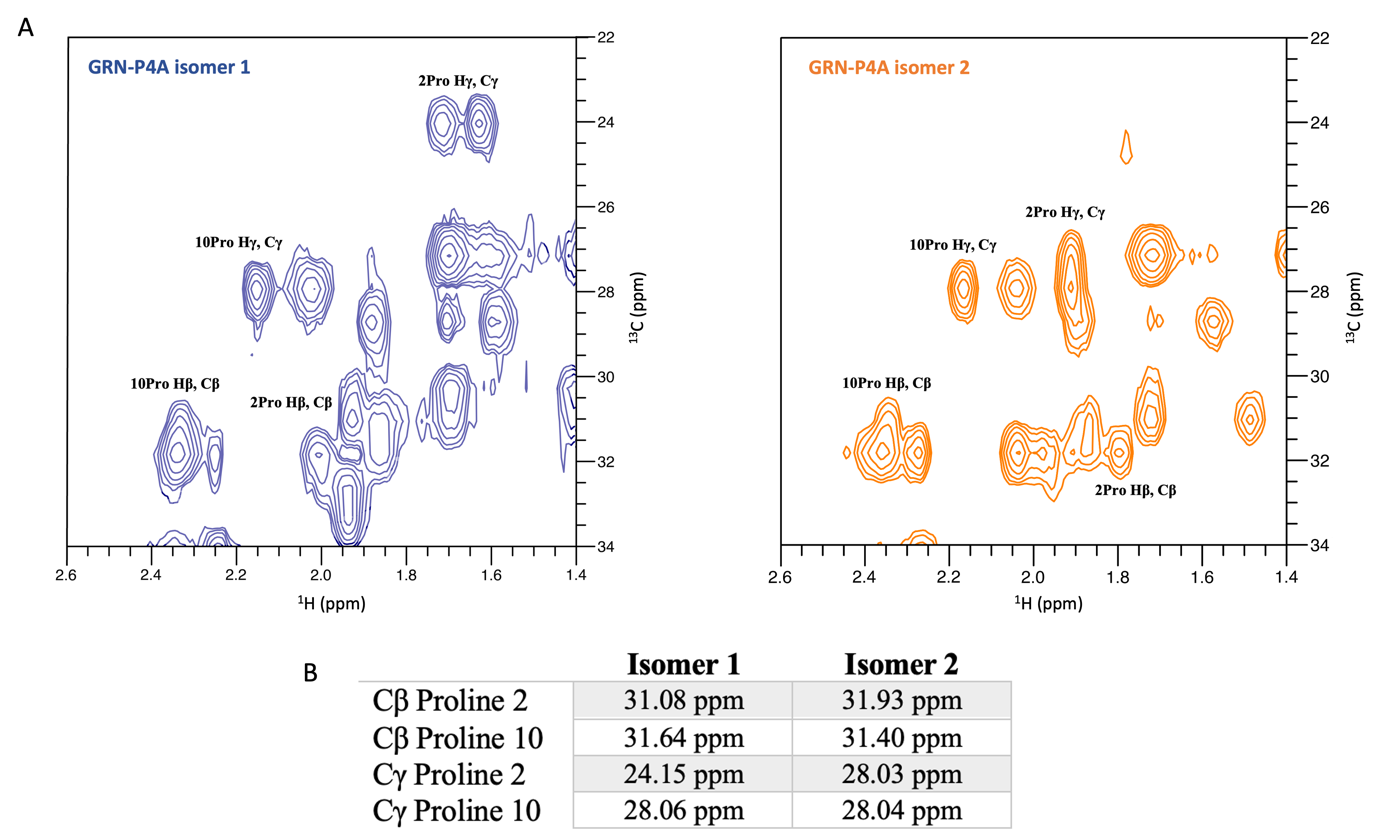


**Figure S5. [^1^H,^13^C]-HSQC NMR spectra and Cβ and Cγ chemical shift table of GRN-P4A isomer 1 and 2.** Samples were dissolved in a solution of 500 μL water and 50 μL D_2_O, for a final peptide concentration of 0.2 mM. The experiments were carried out at 290 K using a 600 MHz Bruker Avance III spectrometer (see Experimental procedures). **(A)** [^1^H,^13^C]-HSQC spectra of isomer 1 (left) isomer 2 (right) annotated proline with resonance assignment. **(B)** Table displaying the chemical shifts for Cβ and Cγ of proline 2 and 10 in isomer 1 and 2.


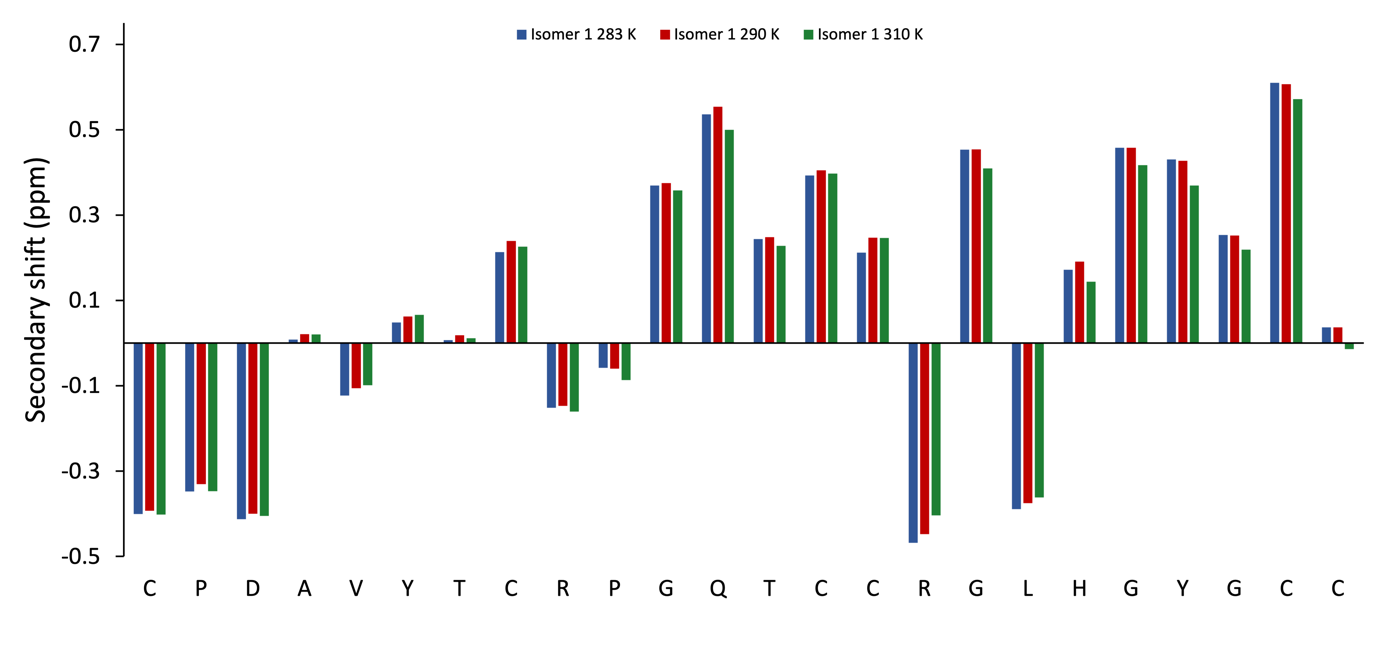


**Figure S6. Comparison of secondary shifts for GRN-P4A isomer 1 at three different temperatures.** The αH secondary shifts were calculated by subtracting the random coil ^1^H NMR chemical shifts previously reported by Wishart et al. (2) from the experimental αH chemical shifts. The peptide displays comparable secondary shift trends across the three temperatures selected for NMR analysis, indicating that no temperature-induced structural changes occur within this range. This finding suggests that isomer 1 maintains a stable three-dimensional conformation and is distinct from isomer 2, with no evidence of interconversion.


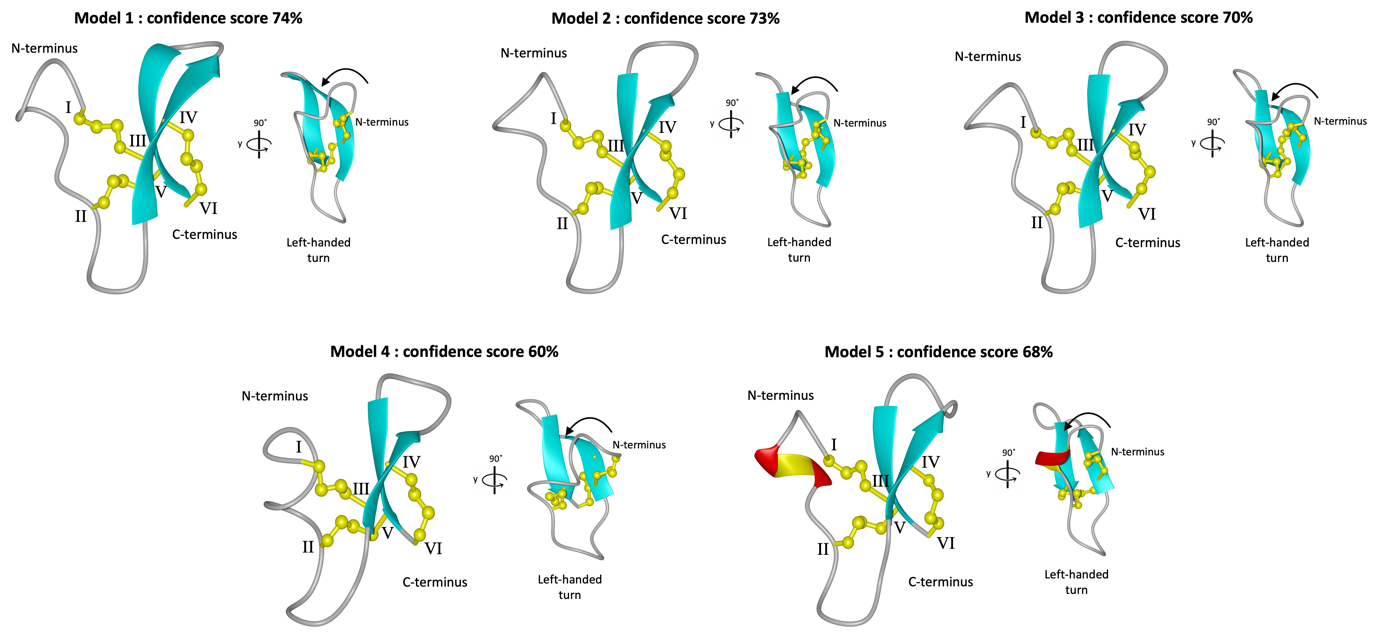


**Figure S7. Ensemble of the five AlphaFold predicted models for GRN-P4A with confidence scores. Three-dimensional structure and disulfide connectivity of the five AlphaFold 3 (3) predictions with the highest confidence scores.** A 90° rotation along the y-axis illustrates the orientation of the N-terminal turn. All predicted models exhibit a left-handed turn, similar to GRN-P4A. **All the predicted models have a left-handed turn as GRN-P4A.** The cysteine residues are labelled using Roman numerals (I-VI), while the disulfide bonds are coloured in yellow, the β-sheets in cyan, and α-helix in red and yellow.


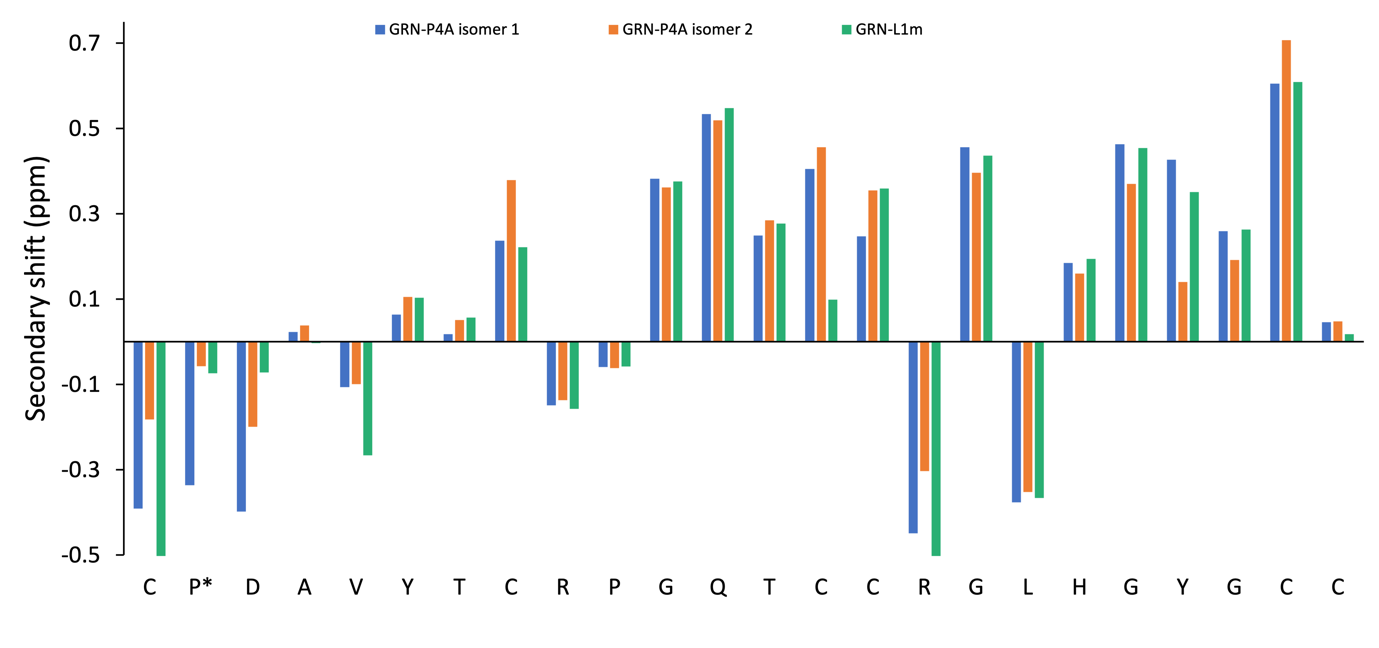


****Figure S8.** Comparison of αH secondary shifts for GRN-P4A isomer 1, GRN-P4A isomer 2 and GRN-L1m.** The αH secondary shifts were calculated by subtracting the random coil ^1^H NMR chemical shifts previously reported by Wishart et al. (2) from the experimental αH chemical shifts. The peptide sequences are shown at the bottom of the diagram. The similarities in the chemical shifts indicate that the peptide adopt the same structural conformations. P* denotes the substitution of proline with a glycine in GRN-L1m.

**References**

1. Chakshusmathi, G., Ratnaparkhi, G. S., Madhu, P. K., and Varadarajan, R. (1999) Native-state hydrogen-exchange studies of a fragment complex can provide structural information about the isolated fragments. *Proc Natl Acad Sci U S A* **96**, 7899-7904

2. Wishart, D. S., Bigam, C. G., Holm, A., Hodges, R. S., and Sykes, B. D. (1995) 1H, 13C and 15N random coil NMR chemical shifts of the common amino acids. I. Investigations of nearest-neighbor effects. *J Biomol NMR* **5**, 67-81

3. Abramson, J., Adler, J., Dunger, J., Evans, R., Green, T., Pritzel, A., Ronneberger, O., Willmore, L., Ballard, A. J., Bambrick, J., Bodenstein, S. W., Evans, D. A., Hung, C. C., O'Neill, M., Reiman, D., Tunyasuvunakool, K., Wu, Z., Zemgulyte, A., Arvaniti, E., Beattie, C., Bertolli, O., Bridgland, A., Cherepanov, A., Congreve, M., Cowen-Rivers, A. I., Cowie, A., Figurnov, M., Fuchs, F. B., Gladman, H., Jain, R., Khan, Y. A., Low, C. M. R., Perlin, K., Potapenko, A., Savy, P., Singh, S., Stecula, A., Thillaisundaram, A., Tong, C., Yakneen, S., Zhong, E. D., Zielinski, M., Zidek, A., Bapst, V., Kohli, P., Jaderberg, M., Hassabis, D., and Jumper, J. M. (2024) Accurate structure prediction of biomolecular interactions with AlphaFold 3. *Nature* **630**, 493-500
